# Fusobacterium nucleatum subsp. polymorphum recovered from malignant and potentially malignant oral disease exhibit heterogeneity in adhesion phenotypes and adhesin gene copy number, shaped by inter-subspecies horizontal gene transfer and recombination-derived mosaicism

**DOI:** 10.1099/mgen.0.001217

**Published:** 2024-03-26

**Authors:** Claire Crowley, Ajith Selvaraj, Arvind Hariharan, Claire M. Healy, Gary P. Moran

**Affiliations:** 1 Division of Oral Biosciences, Dublin Dental University Hospital and School of Dental Science, Trinity College Dublin, Dublin, Ireland; 2 Division of Oral and Maxillofacial Surgery, Oral Medicine and Oral Pathology, Dublin Dental University Hospital and School of Dental Science, Trinity College Dublin, Dublin, Ireland

**Keywords:** adhesins, *Fusobacterium nucleatum*, type V secretion system

## Abstract

*Fusobacterium nucleatum* is an anaerobic commensal of the oral cavity associated with periodontitis and extra-oral diseases, including colorectal cancer. Previous studies have shown an increased relative abundance of this bacterium associated with oral dysplasia or within oral tumours. Using direct culture, we found that 75 % of *Fusobacterium* species isolated from malignant or potentially malignant oral mucosa were *F. nucleatum* subsp. *polymorphum*. Whole genome sequencing and pangenome analysis with Panaroo was carried out on 76 *F*. *nucleatum* subsp. *polymorphum* genomes. *F. nucleatum* subsp. *polymorphum* was shown to possesses a relatively small core genome of 1604 genes in a pangenome of 7363 genes. Phylogenetic analysis based on the core genome shows the isolates can be separated into three main clades with no obvious genotypic associations with disease. Isolates recovered from healthy and diseased sites in the same patient are generally highly related. A large repertoire of adhesins belonging to the type V secretion system (TVSS) could be identified with major variation in repertoire and copy number between strains. Analysis of intergenic recombination using fastGEAR showed that adhesin complement is shaped by horizontal gene transfer and recombination. Recombination events at TVSS adhesin genes were not only common between lineages of subspecies *polymorphum,* but also between different subspecies of *F. nucleatum*. Strains of subspecies *polymorphum* with low copy numbers of TVSS adhesin encoding genes tended to have the weakest adhesion to oral keratinocytes. This study highlights the genetic heterogeneity of *F. nucleatum* subsp. *polymorphum* and provides a new framework for defining virulence in this organism.

## Data Summary

The genome sequence data generated in this study are publicly available from NCBI under BioProjects PRJNA1054149 (Illumina MiSeq Assemblies) and PRJNA1054134 (hybrid Illumina/MinION assemblies). Additional *Fusobacterium* species genome sequences used in this paper have been taken from FusoPortal (http://fusoportal.org/). *F. nucleatum* subsp. *polymorphum* sequences were obtained from GenBank (https://www.ncbi.nlm.nih.gov/datasets/taxonomy/76856/).

**Impact Statement**

*Fusobacterium nucleatum,* a proposed oncobacterium, has been reported to exhibit increased abundance on oral cancers. This study examines the colonization of healthy and diseased mucosa, including sites exhibiting severe dysplasia, and shows for the first time that *F. nucleatum* subsp. *polymorphum* is the most commonly isolated subspecies of *Fusobacterium* on these sites. Bacterial isolates associated with mucosal disease are genetically similar to commensal isolates from the same patient and no disease-associated genotypes were apparent. Analysis of the pangenome of *F. nucleatum* subsp. *polymorphum* revealed a highly recombinogenic organism with a highly variable repertoire of adhesins and putative virulence genes. Variation in the capacity to adhere to an oral keratinocyte cell line was observed with strains possessing few adhesins exhibiting the weakest adhesion patterns. This study highlights the genetic heterogeneity of *F. nucleatum* subsp. *polymorphum* and provides a basis for investigating virulence in this organism.

## Introduction

*Fusobacterium nucleatum* is a rod-shaped, Gram-negative anaerobe, most commonly found in the oral cavity [1]. There are four different subspecies of *F. nucleatum*, namely subsp. *nucleatum*, *polymorphum*, *animalis* and *vincentii* [2,3]. Based on genome sequencing data, some investigators have suggested classifying these as separate *Fusobacterium* species [4,5]. Many studies have provided evidence for *F. nucleatum*’s involvement in periodontitis and in adverse pregnancy outcomes, including preterm labour, stillbirth and chorioamnionitis [6,7]. More recently, *F. nucleatum* has garnered attention as a potential oncobacterium, with increasing evidence showing its links to cancer, particularly colorectal cancer (CRC), but also oral cancer, oesophageal cancer and breast cancer [8,11]. The presence of higher levels of *F. nucleatum* in CRC is also associated with higher mortality, with patients carrying a greater load of the bacterium having a poorer survival rate compared to those with lower or no *F. nucleatum* at the tumour site [12,13]. This may, in part, be due to the fact that *F. nucleatum* has also been shown to promote chemoresistance in CRC [14].

Many studies have reported increased abundance of *F. nucleatum* within oral squamous cell carcinomas (OSCCs) relative to healthy sites, with some noting that *Fusobacterium* was the most abundant genus present in OSCC biopsies and that * F. nucleatum* subsp. *polymorphum* was significantly over-represented in tumours [15,17]. It has also been reported that the abundance of *Fusobacterium* present in the mouth significantly increases with cancer progression from 2.98 % in healthy controls to 4.35 % in stage 1 OSCC and 7.92 % in stage 4 OSCC [18]. Other studies have noted enrichment of *Fusobacteria* and reduced levels of *Firmicutes* on potentially malignant oral lesions such as oral leukoplakia [19].

The virulence landscape at oral tumour sites has also been investigated and it has been found, using meta-transcriptomic analysis, that pathways including iron acquisition, oxidative stress response and peptidase activity were increased at OSCC sites [20]. Here, *Fusobacteria* were reported to be ‘metabolically hyperactive’ at tumour sites, with proteolysis and iron ion transport over-represented. Recently, Galeano Niño *et al*. showed using techniques such as spatial RNA sequencing and FISH that *F. nucleatum* localized to specific micro-niches in OSCC tissues and these areas were associated with localized immunosuppressive effects such as increased expression of ARG1 (arginase 1), the T-cell-inhibitory receptor PD-1 and decreased expression of wild-type p53 [21]. *In vivo* studies in mice, where oral carcinoma was induced using 4-nitroquinoline-1-oxide (4NQO) found that co-infection with *Porphyromonas gingivalis* and *F. nucleatum* enhanced the severity and invasiveness of tumours, with lesions 2.5 times larger in infected mice compared to uninfected mice [22].

*F. nucleatum* possesses an array of adhesive surface proteins, including a range of proteins belonging to the type V secretion system (TVSS), including the well-characterized adhesins Fap2 and RadD [23,24]. Evidence shows that *F. nucleatum* can preferentially bind to Gal-GalNAc residues expressed on cancer cells in CRC and breast cancer via the Fap2 adhesin, enabling the bacterium to colonize tumour sites [11,25, 26]. At the tumour, there have been many mechanisms proposed as to how * F. nucleatum* can impact the progression of cancer, from increased cell proliferation via the interaction with the FadA adhesin, to immune inhibition driven by Fap2 binding to tumour-inhibiting immune cells [27,31]. These findings strengthen the argument that *F. nucleatum*, with its wide range of adhesins, has the potential to be behave as an opportunistic pathogen.

Whole genome sequencing has been employed to study the evolution of *Fusobacterium* using phylogenetics and comparative genomics [32,34]. Many *Fusobacterium* genes are xenologous in origin with high similarity to genes from *Bacteroidetes*, *Proteobacteria*, *Spirochetes* and *Firmicutes*, indicating a high level of horizontal gene transfer (HGT) may have taken place, facilitated by close proximity to other species in the oral cavity [33,35]. More recently, hybrid assemblies of Illumina and MinION sequence reads have facilitated more accurate genome assemblies [36]. Accurate assemblies of type strains of *Fusobacterium* species, including *F. nucleatum* subsp. *nucleatum* (ATCC 23726 and ATCC 25586), *F. periodonticum*, *F. varium*, *F. ulcerans*, *F. mortiferum*, *F. gondiaformans* and *F. necrophorum* subsp. *funduliforme*, are now available at FusoPortal [36]. This interactive resource can allow for deeper investigation and discovery into the genomes of *Fusobacteria*. These assemblies have also proven useful in the characterization of potential virulence factors of *Fusobacterium* species and allowed for the comparison of adhesins, such as TVSS autotransporters, FadA and MORN2 domain-containing proteins, across the different species [37].

The current study aims to apply these genomic techniques to characterize *F. nucleatum* isolates from potentially malignant oral leukoplakia (OLK). We report that *F. nucleatum* subsp. *polymorphum* is commonly isolated from these sites and demonstrate an unforeseen level of heterogeneity in the adhesin gene repertoire of different isolates. We provide evidence that HGT between different subspecies of *F. nucleatum* is highly prevalent and shapes the evolution of adhesins in *F. nucleatum*.

## Methods

### *Fusobacterium* isolate collection and identification

Oral swabs were obtained, with specific consent, from patients with OLK (*n*=25) or OSCC (*n*=2) who were attending the Dublin Dental University Hospital (DDUH). Ethical approval was granted by the Joint Hospitals’ Research Ethics Committee (JREC). Patients who received antibiotics or topical/systemic steroids in the previous 2 months were excluded. OLK was graded as having mild, moderate or severe dysplasia following biopsy by an experienced oral pathologist. A further 11 healthy volunteers attending for routine dental care were sampled at healthy mucosal sites (swabs from lateral border of the tongue or buccal mucosa) and/or supragingival plaque. All mucosal samples (healthy and diseased) were recovered by gentle swabbing of the mucosal surface and were resuspended in 500 µl of STE buffer (Sigma-Aldrich) and 100 µl was plated on Fastidious Anaerobe Agar (FAA) (Neogen), supplemented with 5 % (v/v) defibrinated horse blood (TCS Bioscience) and the antibiotics josamycin (3 µg ml^−1^), vancomycin (4 µg ml^−1^) and norfloxacin (1 µg ml^−1^) (JVN FAA; [38]). These were incubated in a static incubator (Sanyo Electric) at 37 °C in an anaerobic jar (Oxoid) under anaerobic conditions generated with Anaerogen sachets (ThermoFisher Scientific) for 5 days. Colonies with rod-like morphology visualized microscopically were sub-cultured on the FAA JVN media until pure colonies were obtained. Colonies were stored in microbank microbial storage vials (Pro-Lab Diagnostics) at −80 °C.

For preliminary identification, we amplified and sequenced the entire 16S gene. Colonies were resuspended in 10 µl sterile water and heated at 95 °C for 10 min and centrifuged at 16 000 ***g*** for 3 min. One microlitre of the resulting supernatant was used as template in a 25 µl PCR (GoTaq PCR Core System, Promega) with 0.2 µM of each primer (forward 27FYM: 5′-AGAGTTTGATCMTGGCTCAG-3′, reverse 1492R: 5′-ACCTTGTTACGACTT-3′) [39]

PCR products were sequenced by Sanger sequencing (Source Bioscience). Isolates were identified to species by blast searches of the Human Oral Microbiome Database (HOMD) and the National Center for Biotechnology Information (NCBI) database using default parameters (blastn, evalue 1e-05, max_target_seqs 20, sc-match 2, sc-mismatch -3, gap-open 5, gap-extend 2). *F. nucleatum* subsp. *polymorphum* identities were also confirmed by phylogenetic analysis of the core genome alignment, described below.

### Illumina whole genome sequencing

Bacterial cultures were grown anaerobically for 72 h in Brain Heart Infusion (BHI) broth and pelleted by centrifugation at 15 000 ***g*** for 5 min in a bench-top centrifuge. Whole genomic DNA was extracted using the Wizard Genomic DNA kit (Promega), as per the manufacturer’s instructions. Whole genome sequencing of isolates was carried out using the Illumina MiSeq (Illumina). Samples were prepared using the Illumina Nextera DNA Flex library prep and MiSeq V2 (500 cycle) kit, as previously described [40]. *De novo* assembly of Illumina reads was carried out using Unicycler (version v0.4.8), which employed SPAdes to produce fasta format contigs [41,42]. These files were then annotated with Prokka (version 1.14.6), using the annotated genome of *F. nucleatum* subsp. *polymorphum* NCTC10562 as the reference genome [35,43]. The resulting GFF files were analysed with Panaroo (version 1.2.9) using the default settings to determine pangenome structure [44]. The core genome alignment file from this was used to produce a phylogenetic tree using Fastree (version 2.1.10) [45]. Trees were visualized and annotated using iTree of Life [46] or FigTree (version 1.4.4). Principal component analysis (PCA) was performed in Pagoo using a matrix showing gene presence or absence in each strain [47]. Further gene annotation of the pangenome was carried out using EggNOG [48]. Genome-wide association analysis was carried out using SCOARY (version 1.6.16) [49].

### Long-read MinION sequencing

Third-generation long-read sequencing was carried out using the MinION device and SpotON flow cell R9.4.1 from Oxford Nanopore Technologies. Sample preparation was carried out using the genomic DNA sequencing kit SQK-LSK109 and the EXP-NBD104 barcoding kit as per the manufacturer’s instructions. Sequencing was carried out using the MinKnow application (version 3.6.0). After 72 h, raw-read Fast5 files were base called using Guppy base caller and reads were demultiplexed using Guppy barcoder (version 3.2.8).

Hybrid genome assemblies consisting of Illumina short reads (forward and reverse reads) and MinION long reads were assembled for each sample using Unicycler (version v0.4.8) [41]. FastQ files from both platforms were used to perform the assembly. Assemblies were viewed in Bandage (version 0.8.1) to visualize the completed assemblies [50]. Hybrid genomes were annotated with Prokka [43]. Genome comparison files were generated using NCBI-blast+ (version 2.12.0) which were visualized using the Artemis Comparison Tool (ACT, version 18.1.0) [51].

### Identification and annotation of autotransporter genes

The pangenome reference file from Panaroo, containing a list of representative proteins in the pangenome, was interrogated using HMMER3 which uses hidden Markov modelling to identify homology, in this case to conserved autotransporter domain motifs [52,53]. Putative autotransporter gene families identified by HMMER3 were then extracted from the pangenome. These sequences were filtered using SeqKit (version 2.2.0) to remove proteins <100 aa long and SignalP 6.0 to remove any sequences without a signal sequence [54]. The remaining amino acid sequences were used to create a sequence similarity network (SSN) using the EFI Enzyme similarity tool [55]. Similarity networks were then visualized in Cytoscape (version 3.8.0) to delineate closely related networks of proteins. Representative TVSS proteins from *F. nucleatum* subsp. *nucleatum* ATCC 23726 were included as references [36]. Recombination was analysed using fastGEAR (version 9.0.1) [56]. Input files for fastGEAR analysis consisted of nucleotide sequence alignments generated using MAFFT (version 7) [57] and fastGEAR was run using the default settings. The log[BF] refers to the (natural) logarithm of the Bayes factor, a measure of statistical significance based on changes in SNP density between the two lineages. By default, fastGEAR reports ancestral recombinations with BF >10, where it is 10 times more probable that a change in SNP density is a result of a recombination event than not. In ancestral recombination events, based on principles of maximum parsimony, the larger sequence block is assumed to be the donor lineage.

### Adhesion assay

H376 oral keratinocytes, derived from an OSCC from the floor of the mouth, were kindly supplied by Dr Simon Whawell, University of Sheffield, Sheffield, UK. Cells were maintained Keratinocyte Growth Media (KGM) as described previously [58]. For adhesion assays, six-well plates (Greiner CellStar) were seeded with 500 µl of a 5×10^5^ cells ml^−1^ suspension of H376 cells and incubated at 37 ˚C and 5 % CO_2_ for 3–4 days until the cells reached a confluency ~80 % (~1×10^6^ cells per well). * F. nucleatum* subsp. *polymorphum* strains were grown to exponential phase overnight in anaerobic BHI, washed in sterile PBS three times and added to each well at an m.o.i. of 10 : 1. The plate was spun at 800 ***g*** for 5 min and incubated for 2 h at 37 °C in a 5 % CO_2_ incubator. The wells were then washed three times with sterile PBS to remove any unattached bacteria and the epithelial cells were lysed to release adherent bacteria using sterile water. The lysate was serially diluted and plated on blood agar plates and incubated anaerobically at 37 °C for 4–5 days. The plates were then examined for colonies and the colonies enumerated for estimating the percentage of adhesion. The control strain NCTC10562 was included in all assays.

## Results

### Distribution of *F. nucleatum*

**Fig. 1.**
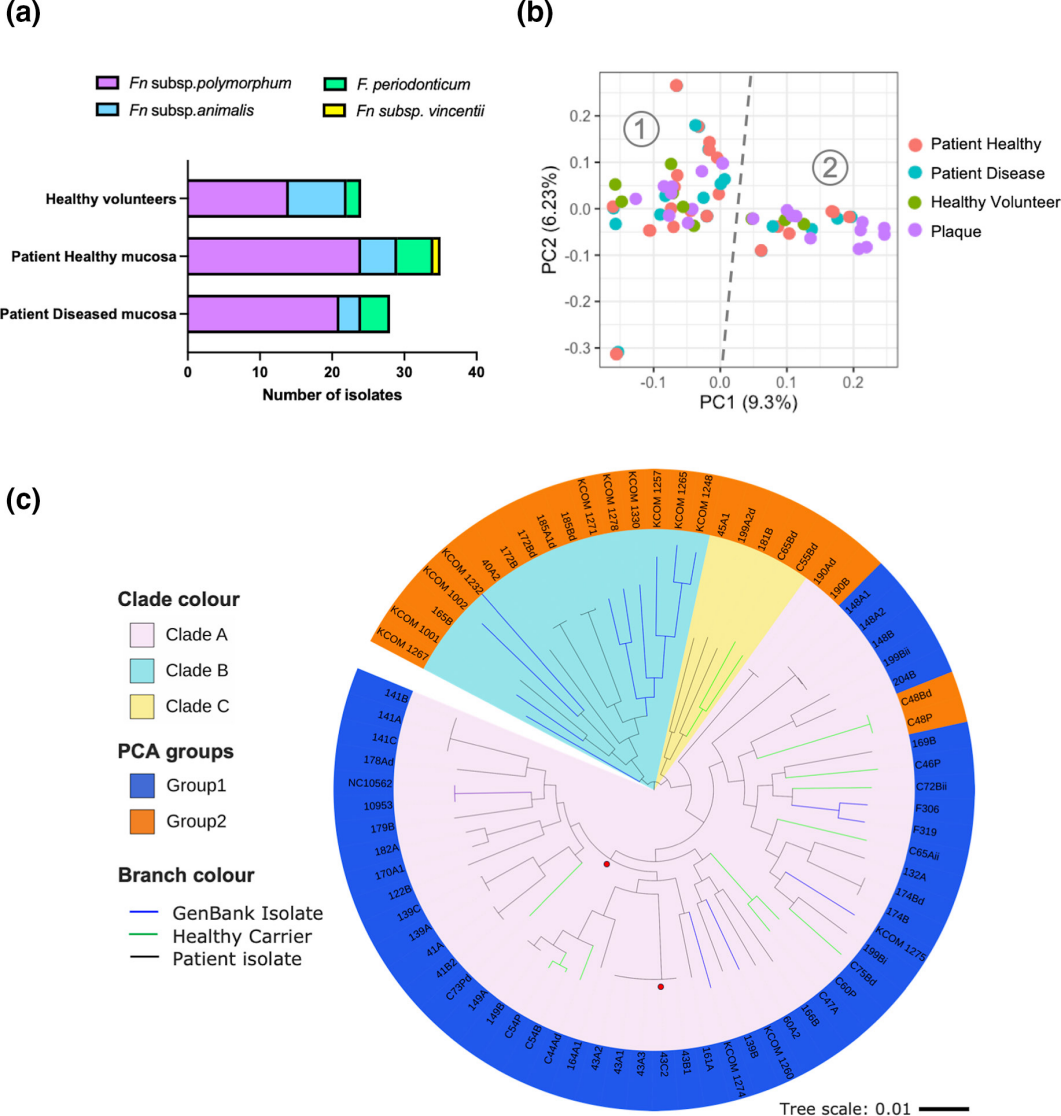
Recovery of *F. nucleatum* subsp. *polymorphum* from the oral cavity. (**a**) Proportion of isolates identified as *F. nucleatum* subsp. *polymorphum* recovered from healthy and diseased mucosa. (**b**) Distribution of *F. nucleatum* subsp. *polymorphum* isolates based on PCA of a gene presence/absence matrix for each strain. The dotted line indicates separation of the population into two groups along axis 1, with group 1 on the left and group 2 on the right. (**c**) Maximum likelihood phylogenetic tree generated from the core genome alignment of *F. nucleatum* subsp. *polymorphum* isolates showing three main clades and the two PCA groups from (**b**). All branches have bootstrap values >99 % except two, indicated by red circles. Tree scale indicates degree of nucleotide sequence divergence.

Patients attending the Dublin Dental University Hospital were sampled for the presence of oral *Fusobacterium* spp. by direct culture on FAA JVN media (Table S1, available in the online version of this article). Of the patients sampled, 25 were attending for potentially malignant OLK and two patients were diagnosed with OSCC. Patients were sampled by swabbing both the site of mucosal disease and a contralateral healthy mucosal site (Table S1). A further 11 healthy volunteers attending for routine dental care were sampled at healthy mucosal sites (swabs from lateral border the tongue or buccal mucosa) and/or supragingival plaque in order to investigate if the same genotypes of *F. nucleatum* could be recovered from mucosa and plaque in the same individual (Table S1). We recovered 87 isolates of *Fusobacterium* species from these individuals and following sequence analysis of the 16S rRNA gene 59 isolates (67.8 %) could be classified as *F. nucleatum* subsp. *polymorphum*, followed by *F. nucleatum* subsp. *animalis* (*n*=16, 18.4 %), *F. periodonticum* (*n*=11, 12.65 %) and a single isolate of *F. nucleatum* subsp. *vincentii* (*n*=1, 1.15 %; Fig. 1a). Confirmation of the isolate identities was obtained following whole genome sequencing and phylogenetic analysis of a core set of 173 *Fusobacterium* genes, which showed that our collection of *F. nucleatum* subsp. *polymorphum* formed a distinct clade separate from other subspecies of *F. nucleatum* (Fig. S1). *F. nucleatum* subsp. *polymorphum* represented 75 % of the isolates recovered from OLK (*n*=21), including mild dysplasia (*n*=8), moderate dysplasia (*n*=5), severe dysplasia (*n*=7) and OSCC (*n*=1) (Fig. 1a; Table S1). We also recovered *F. nucleatum* subsp. *polymorphum* from contralateral healthy mucosa in these patients where this species represented 70 % of isolates recovered (Fig. 1a; Table S1). In healthy volunteers without mucosal disease, *F. nucleatum* subsp. *polymorphum* represented a smaller proportion of the total isolates, 58 % (14/24), and was recovered from mucosal surfaces (*n*=9) and from plaque (*n*=5) with three subjects yielding isolates from both areas (Fig. 1a; Table S1). The remaining isolates recovered were identified as *F. periodonticum* and *F. nucleatum* subsp. *animalis*, with the latter found more commonly in healthy participants (33 %) compared to patients with mucosal disease (12 %; Fig. 1a). Differences in species distribution in Fig. 1a were not significant (Fisher’s exact test *p*=0.37). Supragingival plaque samples that were culture positive for *Fusobacterium* (n=7) exhibited a similar distribution of subspecies *polymorphum* (n=5) and *animalis* (n=2).

### The pangenome of *F. nucleatum* subsp. *polymorphum*

As the most commonly recovered *F. nucleatum* subspecies, we further characterized the pangenome of *F. nucleatum* subsp. *polymorphum*. Whole genome sequencing and pangenome analysis with Panaroo was carried out on 76 isolate genomes, including 60 genomes sequenced in this study and 16 assemblies downloaded from GenBank including the reference strain ATCC 10953. GenBank sequences included isolates recovered from dental plaque from Korean subjects reported by Kim *et al*. [2]. Accurate, single chromosome, hybrid genome assemblies combining long MinION reads and short Illumina reads were generated for the type strain NCTC10562 and four additional clinical strains (40A2, C48P, 43A3 and 149A).

**Fig. 2.**
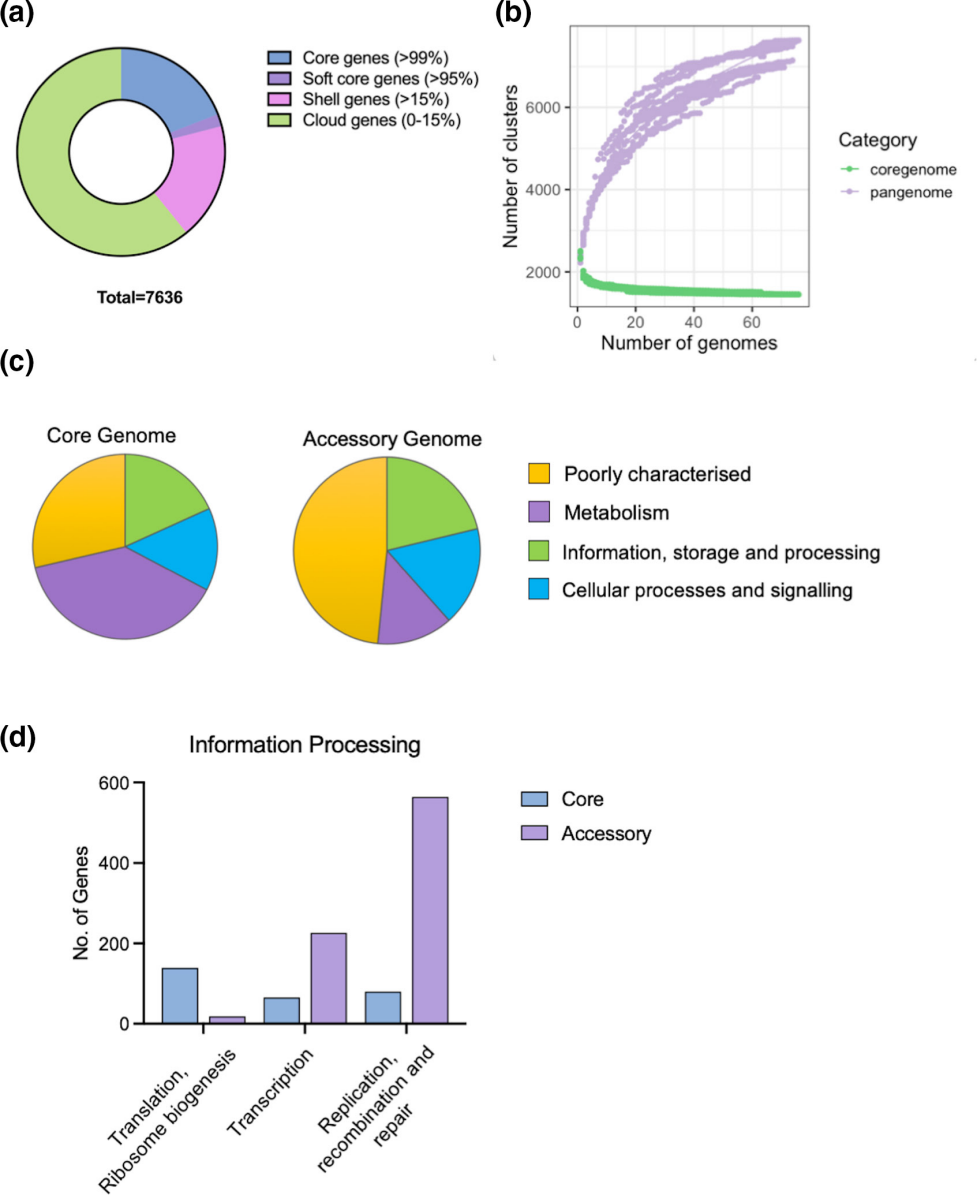
The pangenome of *F. nucleatum* subsp. *polymorphum*. (**a**) Distribution of genes from the 76 genomes into core, shell and cloud (accessory) genomes. (**b**) Rarefaction curve analysis of the pangenome. (**c**) Classification of genes by COG categories using EggNOG. (**d**) Comparison of subcategories of the genes in the ‘Information, storage and processing’ category in the core and accessory genomes.

Pangenome analysis of the collection in Panaroo identified a relatively small core genome of 1604 genes in 95 % of strains in a total pangenome of 7636 genes (Fig. 2a, Table S2). Rarefaction curve analysis of the accessory genome indicates an open pangenome structure with many individual isolates possessing strain-specific genes (Fig. 2b). Annotation of the core genome using EggNOG indicated that this gene set was dominated by metabolic functions which were a minor component of the accessory genome (Fig. 2c, Tables S3 and S4). In the core genome, the category ‘Information processing and storage’ was dominated by genes encoding factors required for transcription, translation and DNA replication, whereas in the accessory genome, this category was rich in transposases and helix–turn–helix proteins (Fig. 2d). EggNOG also determined that 98 % of genes in the core genome had strongest homology to orthologous genes from *Fusobacteria*, whereas in the accessory genome only 70 % of genes had a closest orthologue in *Fusobacteria*, with 7 % having a closest orthologue in *Clostridia* and 1.7 % in *Bacilli*.

To investigate the population structure of *F. nucleatum* subsp. *polymorphum*, we carried out PCA of the pangenome (Fig. 1b) and also reconstructed a phylogenetic tree based on the alignment of the core genomes of the isolates generated by Panaroo (Fig. 1c). PCA divided the population into two main groups along axis 1 (Fig. 1b). Phylogenetic analysis delineated three main clades of strains, with the biggest clade A (*n*=55) largely corresponding to PCA group 1. Clades B (*n*=16) and C (*n*=5) were largely made up of strains from the smaller PCA group 2. Each clade and PCA group contained strains from diseased and healthy mucosa suggesting that no single genotype is associated with mucosal disease (Fig. S2). Clade B contained a significant number of the Korean plaque-derived isolates (10/16).

A total of 12 patients were sampled at multiple locations, including OLK and a contralateral healthy mucosal site. In 10 of these 12 individuals, the phylogenetic tree shows that isolates from healthy and OLK sites cluster together indicating a high degree of genetic similarity. In the case of two healthy participants (C54 and C48), genetically similar isolates could be recovered from plaque and mucosal surfaces (Fig. 1c). Exceptions to this include patient 199, from whom an OLK isolate (199A2d) and two isolates from healthy mucosa (199Bi and 199Bii) were recovered, none of which clustered together on the tree showing that multiple strain carriage is also possible (Fig. 1c). Similarly, in the case of the isolates from patient 139, isolate 139B (healthy mucosa, lateral tongue) was genetically unrelated to isolates 139A (OLK, ventral tongue) and 139C (healthy buccal mucosa) (Fig. 1c, Table S1).

Genome-wide association studies were carried out to determine if any genes were specific to each PCA group. Notably, most PCA group 2 isolates (15/25) were found to possess an ornithine catabolism operon which was absent in PCA group 1 isolates (Fig. S3).

### Adhesin gene analysis

We analysed the pangenome to determine the distribution of genes with known or putative virulence functions, specifically autotransporters belonging to the TVSS and genes with homology to the putative adhesin encoding gene *fadA*.

**Fig. 3.**
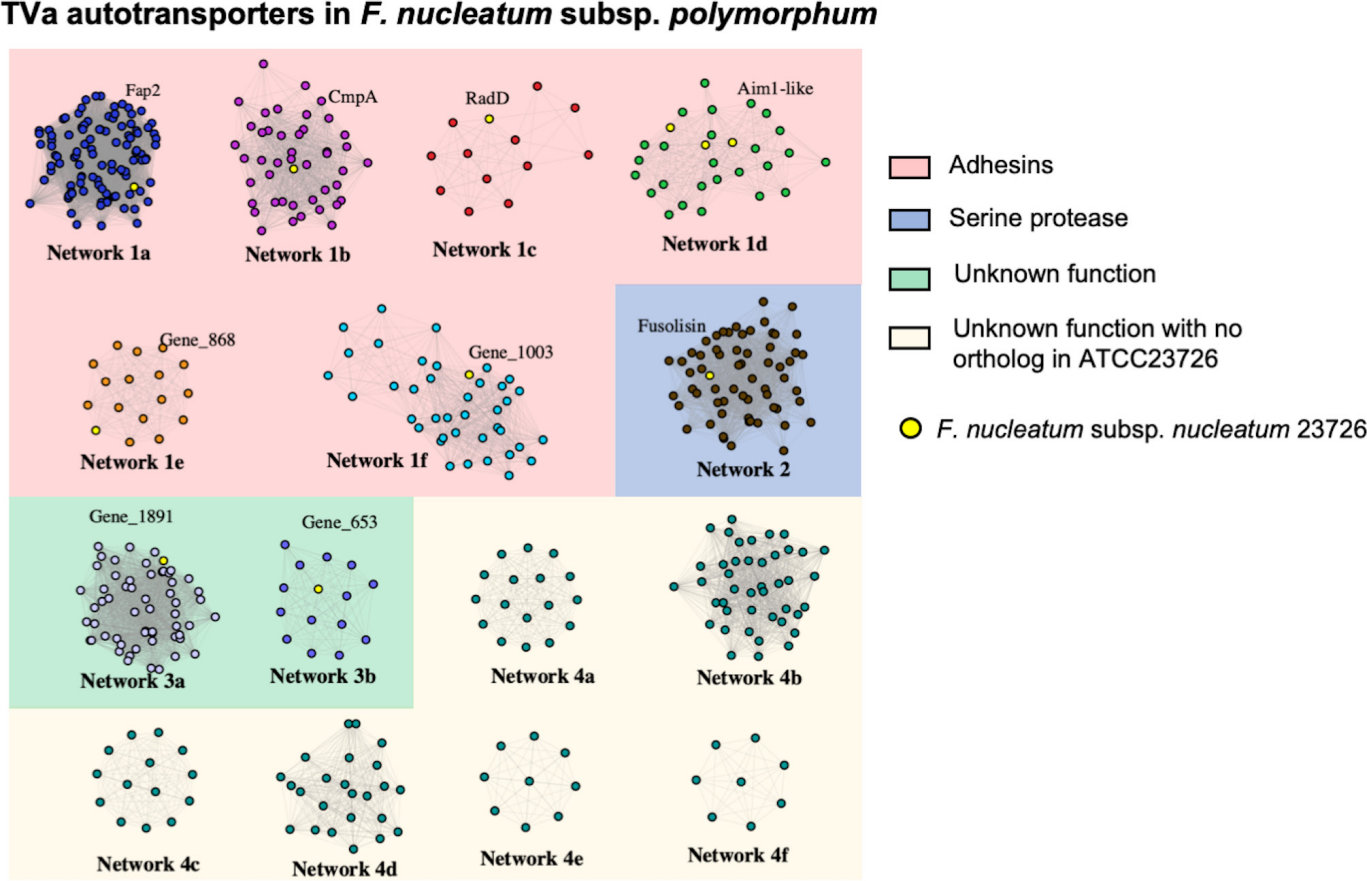
Classification of type Va secretion system (TVaSS) proteins in *F. nucleatum* subsp. *polymorphum*. Separate networks represent individual homology clusters of proteins generated using the EFI Enzyme similarity tool. Network identity was inferred from homology to genes from *F. nucleatum* subsp. *nucleatum* ATCC 23726 including putative adhesins (Networks 1a–1f), serine proteases (Network 2) and proteins of unknown function (Networks 3 and 4). The Aim1-like cluster includes Aim1, Gene_351 and Gene_665 from strain 23726.

The TVSS proteins can be further classified into subtypes TVa–TVe [59]. Panaroo identified multiple homology groups with similarity to the TVa autotransporters and to further characterize their relatedness, we interrogated the set of translated proteins from the pangenome using HMMER searches, which uses hidden Markov modelling to identify homology. The resulting sequences were used to create networks using the EFI Enzyme similarity tool and visualized in Cytoscape to identify closely related networks of proteins (Fig. 3). Representative TVa proteins from *F. nucleatum* subsp. *nucleatum* ATCC 23726 were included as references. In the case of the TVa proteins, we could detect 15 groups of proteins, including networks with similarity to the TVa adhesins Fap2, RadD, CmpA and Aim1 (Networks 1a–1d; Fig. 3). Network 2 included serine proteases similar to fusolisin, Network 3a included orthologues of Gene_1891 from strain ATCC 23726 and Network 3b included orthologues of Gene_653 from strain ATCC 23726 (Fig. 3). Networks 4a to 4f had no direct orthologues in ATCC 23726 (Fig. 3). The sequences of all identified TVa proteins can be found in the supplementary material.

**Fig. 4.**
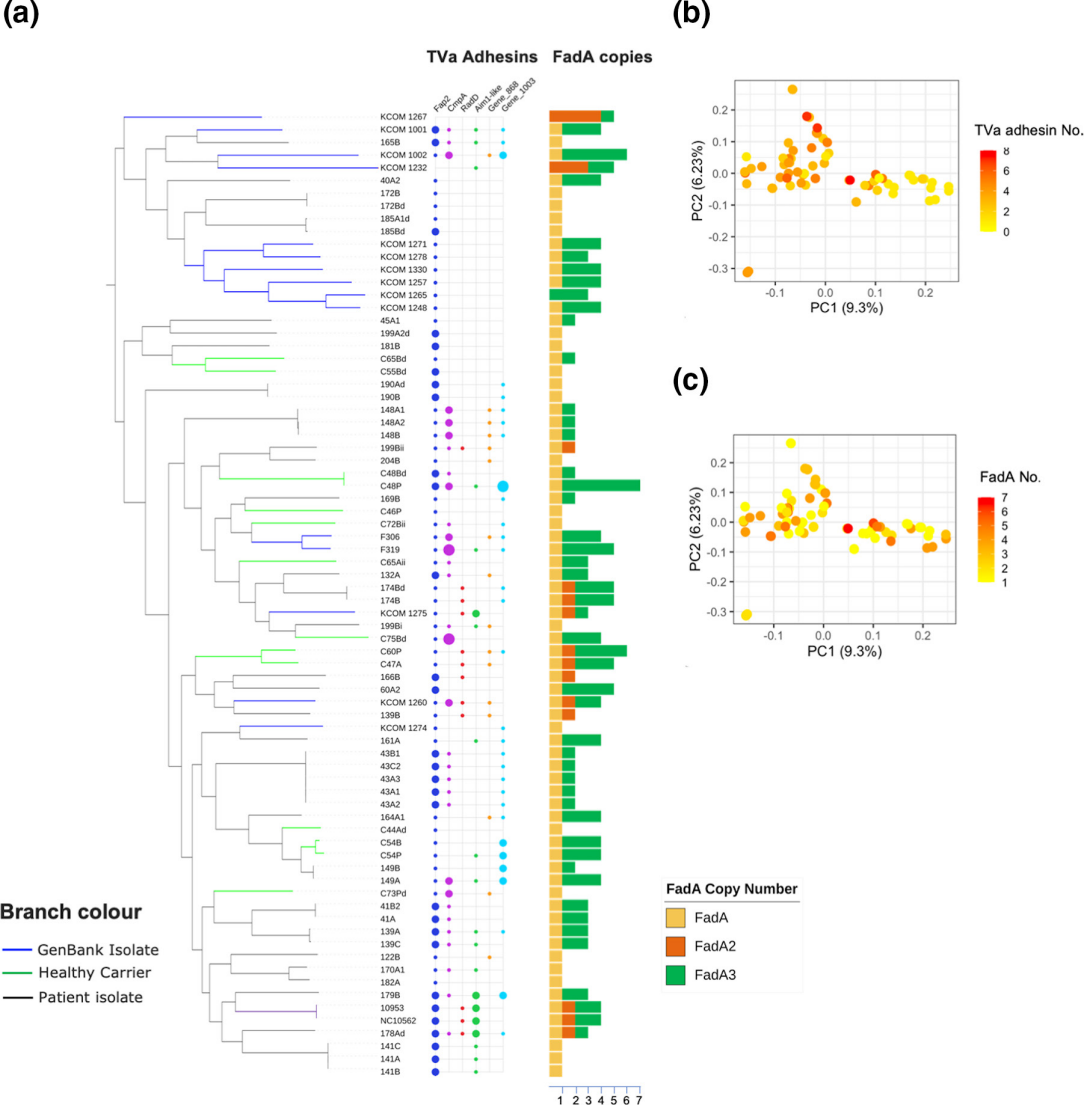
Analysis of adhesin copy number in the *F. nucleatum* subsp. *polymorphum* population. (**a**) Maximum likelihood phylogenetic tree based on the core genome alignment showing the relationship between adhesin copy number and genotype. Coloured circles indicate the presence of the indicated TVa adhesins in each strain, with circle diameter indicating adhesin gene copy number (ranging from one to three). Bar plot on the right indicates copy number of FadA adhesin encoding genes, with *fadA* (yellow) present as a single copy in most strains. (**b**) PCA of the pangenome based on a gene presence/absence matrix, colour coded to show copy number of TVa adhesins in each strain. (**c**) PCA plot of pangenome structure showing copy number of FadA adhesins in each strain.

Fap2 is the most common Type Va adhesin present in the pangenome, with all isolates except for KCOM_1232 and KCOM_1267 possessing at least one copy, and with 29 of the isolates encoding a second paralogous gene (Fig. 4a). The Fap2 protein exhibits variation in size, from 3732 to 4661 aa. In the case of *cmpA*, 31 isolates have at least one of copy of this adhesin, with 20 of these possessing a single copy, nine possessing two copies and two possessing three copies (Fig. 4a). Aim1-like adhesins are found in 20 isolates, with 15 possessing single copies and five with two copies (Fig. 4a). Other unnamed and uncharacterized adhesins orthologous to Gene_868 and Gene_1003 in ATCC 23726 were also found in our isolates, with 16 and 31 isolates, respectively, possessing at least one copy of these adhesins (Fig. 4a). RadD is the least common adhesin found among the isolates, present only in 12 isolates (Fig. 4a). Of these, three have truncated versions of the adhesin (174B, 174Bd and KCOM_1260) where it is less than 1600 aa long, compared to the other isolates, where the length varies from 3534 to 3588 aa.

Overall, the number of TVa adhesins possessed by our isolates varies greatly from isolate to isolate, with numbers ranging from one to eight, with one isolate possessing no intact genes (KCOM_1267) (Fig. 4a). When TVa adhesin copy number was compared to strain genotype, we observed that PCA group 2 isolates tended to have fewer TVa adhesin coding genes compared to PCA group 1 isolates (Fig. 4b).

The presence of Type Vb autotransporters showed less variability, with most isolates possessing between one and three copies. The Type Vc autotransporters can be further broken down to TVcA–TVcE. While each type of these TVc autotransporters can be found in ATCC 23726, only TVcE was found in our *F. nucleatum* subsp. *polymorphum* collection, in single copies except for two isolates (KCOM_1232 and F306). The Type Vd autotransporter in *F. nucleatum* is a phospholipase called FplA and was found to be present in single copies in all of the isolates [60].

### Distribution of FadA genes

In the case of FadA, a single copy of this adhesin is present in all isolates except for KCOM_1232, KCOM_1265 and KCOM_1267 (Fig. 4a). The other members of the FadA family are more varied in their distribution among the isolates. FadA2 is present only in 14 isolates, usually as a single copy within an operon with *radD*. Exceptions to this included strains KCOM_1232 and KCOM_1267 which have three and four copies, respectively (Fig. 4a). FadA3 is the most varied in terms of copy number. It is present in 49 isolates, with copy numbers ranging from one to six. It is interesting to note that in ATCC 23726, the FadA3 proteins (FadA3a–c) are 131 aa long and are gene triplications, with 100 % identity at the amino acid level. In *F. nucleatum* subsp. *polymorphum* the multiple copies of FadA3 exhibit variation in both amino acid sequence and length (Fig. S4). There are two variants of the protein, one 131 aa long and the other 134 aa long. Seven isolates possess only the shorter version, 24 possess only the longer variant, while both types are found in 15 isolates. FadA copy number was not related to genotype (Fig. 4c).

### Comparison of gene order and synteny

**Fig. 5.**
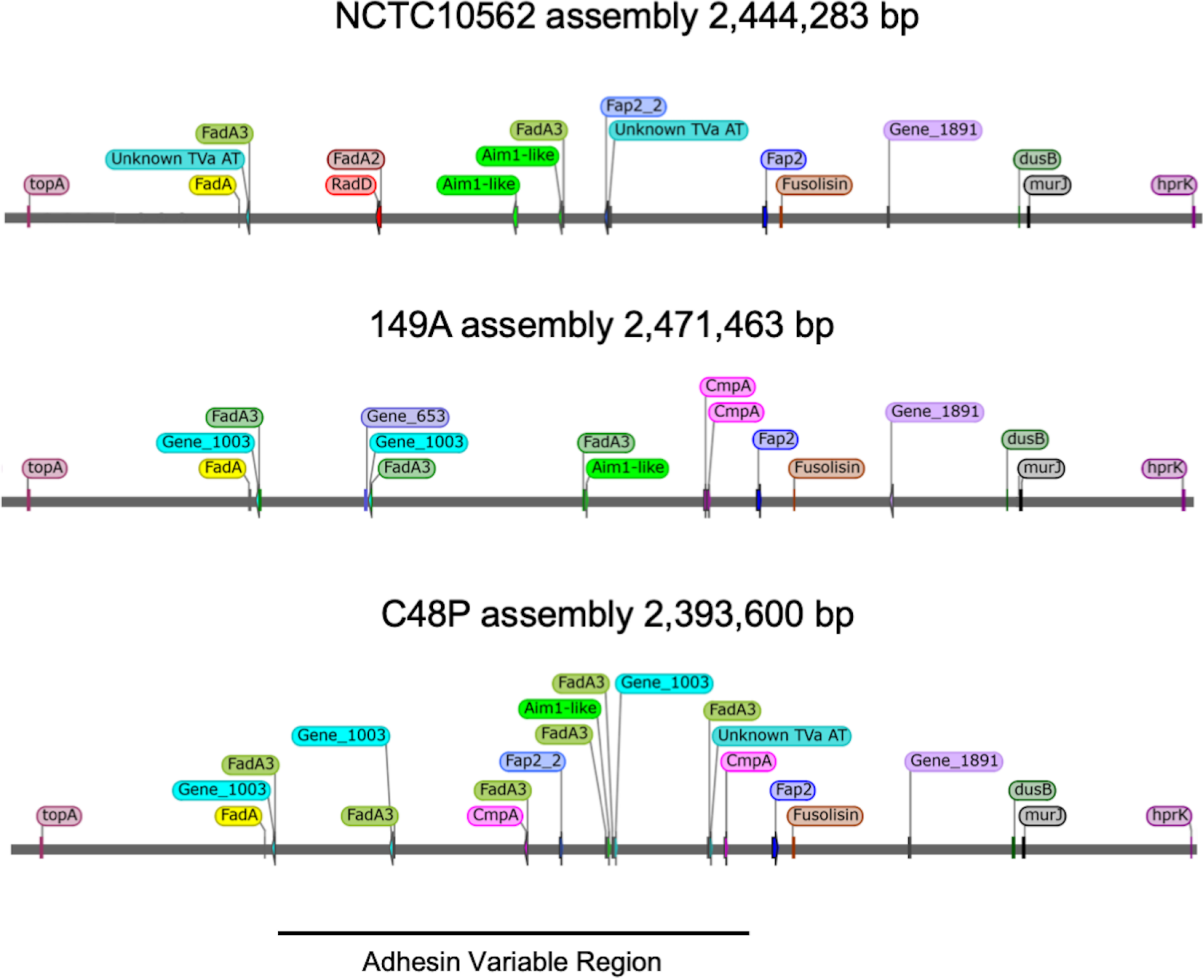
Comparison of synteny and genomic location of TVa adhesin encoding genes and *fadA* orthologues in hybrid genome assemblies. Annotation of hybrid genome assemblies was carried out using Prokka and identification of the TVa autotransporter and FadA encoding genes was carried out by HMMER searches with conserved motifs. The line below indicates the region of the genome exhibiting variation in adhesin gene complement. Linear genome representations were generated using SnapGene viewer (snapgene.com).

The genomes which underwent hybrid assembly were used to compare the synteny and genomic location of TVa adhesin encoding genes and *fadA* orthologues. Despite differences in adhesin gene complement between strains, we observed a general conservation of synteny across the chromosome, with *fadA*, *fusolisin* and *fap2* having conserved locations (Fig. 5). The region between the *fadA* and *fap2* genes exhibits the greatest variation and exhibits differences in TVa adhesin repertoire and copy number (Fig. 5). It was also noted that *fadA* genes are commonly co-located with TVa adhesin genes (Fig. 5).

Closer examination of the *fap2* locus shows high conservation of gene order in *F. nucleatum* subsp. *polymorphum* (Fig. S5). Comparison with the *fap2* locus in subspecies *nucleatum, animalis* and *vincentii* showed that gene order upstream of *fap2* is also conserved in these subspecies (Fig. S5). Where present, the *radD* operon (which includes *fadA2*) was found to be located at a conserved region downstream of *secY* in all *F. nucleatum* subsp. *polymorphum* isolates (Fig. S6). In isolates where *radD* is absent, the whole operon is missing, but synteny of the surrounding genomic region is conserved (Fig. S6). The *radD* operon was found to be located at a different genomic location in subspecies *nucleatum, animalis* and *vincentii* (Fig. S6).

### Recombination and HGT shapes TVa adhesin sequences

Phylogenetic analysis of the TVa adhesins revealed that some strains of *F. nucleatum* subsp. *polymorphum* possess adhesins that clustered together with genes from *F. nucleatum* subsp. *nucleatum,* rather than orthologous genes from within the subsp. *polymorphum* pangenome (Fig. S7). As this suggested recombination or HGT events between different subspecies, we investigated this further by analysing the nucleotide sequence alignments of TVSS autotransporters using fastGEAR to detect HGT events and recombination-derived mosaicism. We included sequences from subspecies *nucleatum*, *animalis* and *vincentii* and from *F. periodonticum* for these analyses. We initially analysed the sequences of several housekeeping genes, namely *gyrB, recA, ruvB, ftsZ* and *rsml*, to determine the level of recombination in non-autotransporter encoded genes (Figs S8–S17). In these experiments, genes from subspecies *nucleatum*, *animalis* and *vincentii* generally separated into separate lineages distinct from the orthologous genes from subspecies *polymorphum*. In each case, genes from *F. nucleatum* subsp. *polymorphum* represent a separate and distinct lineage compared to the other subspecies (Figs S8–S17). Recombination events appear to be rare in these housekeeping genes, the main exception being *gyrB* which exhibited evidence of recent recombination between genes in the subspecies *animalis*, *polymorphum* and *vincentii* lineages (Figs S8 and S9).

**Fig. 6.**
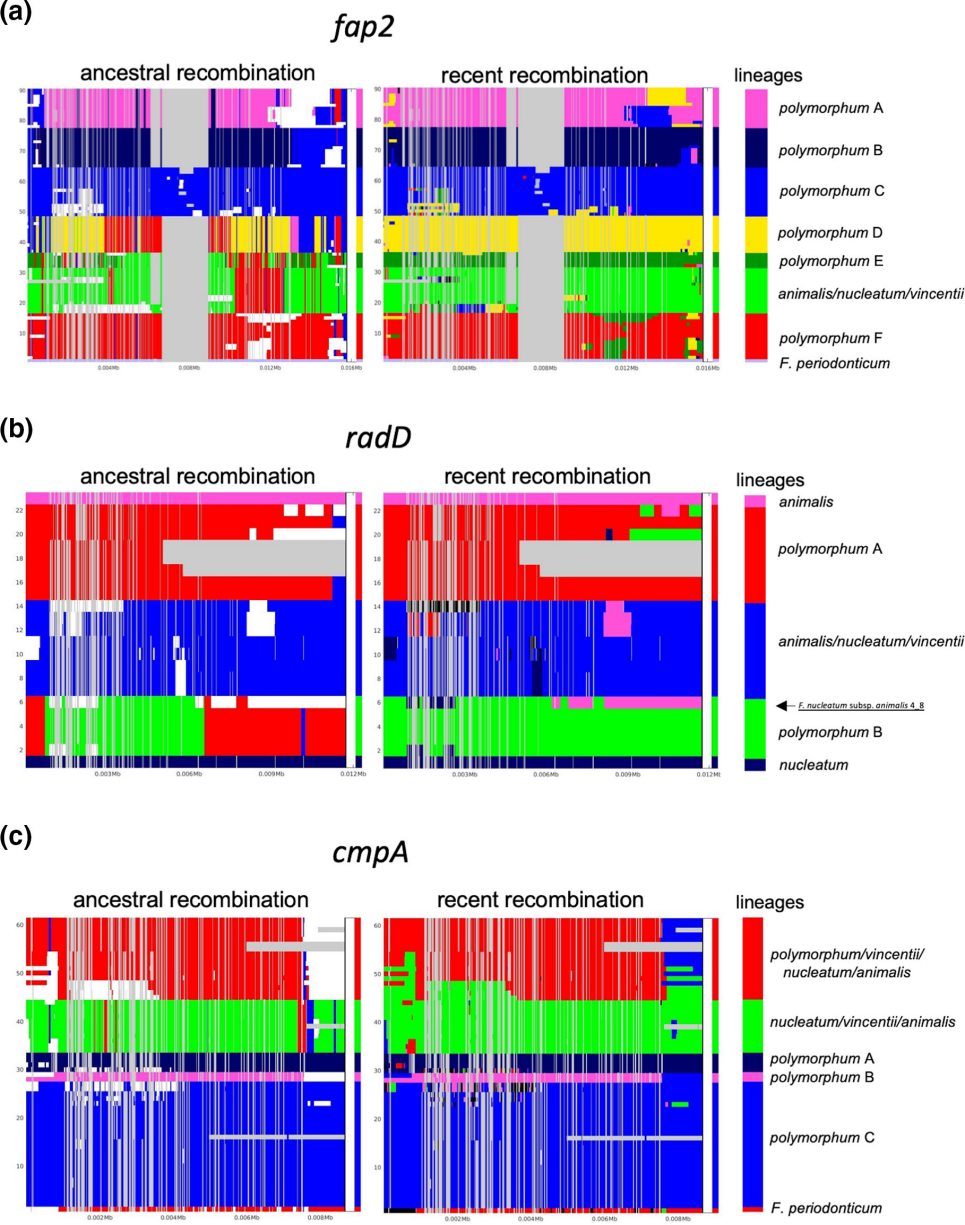
Analysis of recombination in *Fusobacterium* species using fastGEAR. Plots correspond to sequence alignments of (**a**) *fap2* (*n*=90), (**b**) *radD* (*n*=23) and (**c**) *cmpA* (*n*=61). Sequences included on the *y*-axis are ordered by a phylogenetic tree. The *x*-axis corresponds to the sequence positions of the respective genes. The key on the right side of the panel shows division of the strains into lineages indicated by different colours and named according to the subspecies represented within. Grey sections represent gaps in the sequence alignment. White sections in the ancestral panel are recent recombination events which can be seen in the right panel. Phylogenetic trees were generated from nucleotide sequence alignments generated in MAFFT and visualized using FigTree and are shown in Figs S18–S24.

Analysis of the TVa adhesin proteins revealed strong evidence for inter-subspecies HGT and recombination-derived mosaicism. Analysis of *fap2* alignments identified eight distinct lineages, including one lineage for *F. periodonticum*, one combined lineage for the subspecies *nucleatum*, *animalis* and *vincentii* genes, and seven distinct lineages for *fap2* in subspecies *polymorphum* (labelled A–F) (Figs 6 and S18). These analyses showed high levels of ancestral recombination (i.e. inter-lineage) not only within subspecies *polymorphum* lineages, but also between the *nucleatum*/*animalis*/*vincentii* lineage and the subspecies *polymorphum* lineages. Significant recombination (indicated by log[BF]>5.0) was detected between the *nucleatum*/*animalis*/*vincentii* lineage and *polymorphum* lineages C, D, E and F, with the *nucleatum*/*animalis*/*vincentii* lineage possibly acting as both a donor and a recipient (Table 1). Recombination events were detected throughout the gene sequence, including the 3′ end encoding the conserved autotransporter domains and the 5′ end, encoding potential adhesin domains. Analysis of potentially more recent recombination events (recombination within lineages) also identified regions within the *nucleatum*/*animalis*/*vincentii* lineage which may have undergone recombination with subspecies *polymorphum* lineages C, D, E and F (Figs 6 and S19). Analysis of the adhesins *radD* and *cmpA* also highlighted further evidence of recombination between subspecies *polymorphum* and the *nucleatum*, *animalis* and *vincentii* lineages (Figs 6, S20 and S23). In the case of *radD*, an allele from *F. nucleatum* subsp. *animalis* clustered in the *polymorphum* lineage B, exhibiting a high level of mosaicism (Fig. 6b). Similarly, in the case of *cmpA*, a lineage was identified containing alleles from all of the subspecies analysed (*polymorphum*, *nucleatum*, *animalis* and *vincentii,* Fig. 6c, red lineage), again indicating inter-subspecies HGT events. To determine if this level of recombination was only observed in the TVa adhesins, we also examined a TVa effector gene (fusolisin) and a TVc gene (*fvcE*). In the case of fusolisin, we identified ancestral gene lineages which, although dominated by genes from subspecies *polymorphum*, also contained genes from subspecies *nucleatum* (including strain 23726) and subspecies *vincentii*, indicating HGT events between the subspecies (Figs S24 and S25). Analysis of *fvcE* also highlighted recombination events between *F. nucleatum* subspecies, indicating that HGT and mosaicism is not restricted to the adhesin encoding genes (Figs S26 and S27).

**Table 1.** Significant ancestral recombination events (log[BF]>5.0) within the *fap2* gene sequences in the *F. nucleatum* pangenome identified using fastGEAR

Start	End	Lineage 1	Lineage 2	Log(BF)
830	907	*animalis/nucleatum/vincentii*	*polymorphum E*	5.6
1103	1182	*animalis/nucleatum/vincentii*	*polymorphum E*	24.5
3823	3885	*animalis/nucleatum/vincentii*	*polymorphum E*	6.7
4424	4611	*animalis/nucleatum/vincentii*	*polymorphum E*	15.8
4759	9435	*animalis/nucleatum/vincentii*	*polymorphum E*	625.4
9479	9615	*animalis/nucleatum/vincentii*	*polymorphum E*	15.4
9646	9740	*animalis/nucleatum/vincentii*	*polymorphum E*	12.6
10 177	10 282	*animalis/nucleatum/vincentii*	*polymorphum E*	8.7
10 903	10 972	*animalis/nucleatum/vincentii*	*polymorphum E*	14.1
11 589	11 723	*animalis/nucleatum/vincentii*	*polymorphum E*	14.9
11 891	12 244	*animalis/nucleatum/vincentii*	*polymorphum E*	38.2
12 390	12 469	*animalis/nucleatum/vincentii*	*polymorphum E*	19.9
12 793	13 039	*animalis/nucleatum/vincentii*	*polymorphum E*	38.4
14 269	14 438	*animalis/nucleatum/vincentii*	*polymorphum E*	20
14 514	14 847	*animalis/nucleatum/vincentii*	*polymorphum E*	46.8
15 643	15 772	*animalis/nucleatum/vincentii*	*polymorphum E*	34.5
746	779	*animalis/nucleatum/vincentii*	*polymorphum D*	6.9
1192	1212	*animalis/nucleatum/vincentii*	*polymorphum D*	18.1
2461	2499	*animalis/nucleatum/vincentii*	*polymorphum D*	10.3
2652	2685	*animalis/nucleatum/vincentii*	*polymorphum D*	8.6
11 497	11 541	*animalis/nucleatum/vincentii*	*polymorphum D*	5.2
12 182	12 207	*animalis/nucleatum/vincentii*	*polymorphum D*	6
686	791	*polymorphum F*	*animalis/nucleatum/vincentii*	26.6
3957	4030	*polymorphum F*	*animalis/nucleatum/vincentii*	10.8
4092	4266	*polymorphum F*	*animalis/nucleatum/vincentii*	7.6
10 248	11 440	*polymorphum F*	*animalis/nucleatum/vincentii*	72
11 456	11 778	*polymorphum F*	*animalis/nucleatum/vincentii*	12.4
11 825	12 649	*polymorphum F*	*animalis/nucleatum/vincentii*	55.2
13 704	13 748	*polymorphum F*	*animalis/nucleatum/vincentii*	6.2
14 460	14 513	*polymorphum F*	*animalis/nucleatum/vincentii*	11.2
12 650	12 684	*polymorphum C*	*animalis/nucleatum/vincentii*	5.1
15 346	15 396	*polymorphum C*	*animalis/nucleatum/vincentii*	7.2

Finally, analysis of *fadA* indicated the presence of three lineages representing *F. periodonticum*, a subspecies *polymorphum* lineage and a single lineage for *animalis/nucleatum/vincentii,* with no evidence of recombination (Figs 5c, S28 and S29).

### Analysis of adhesion phenotypes

**Fig. 7.**
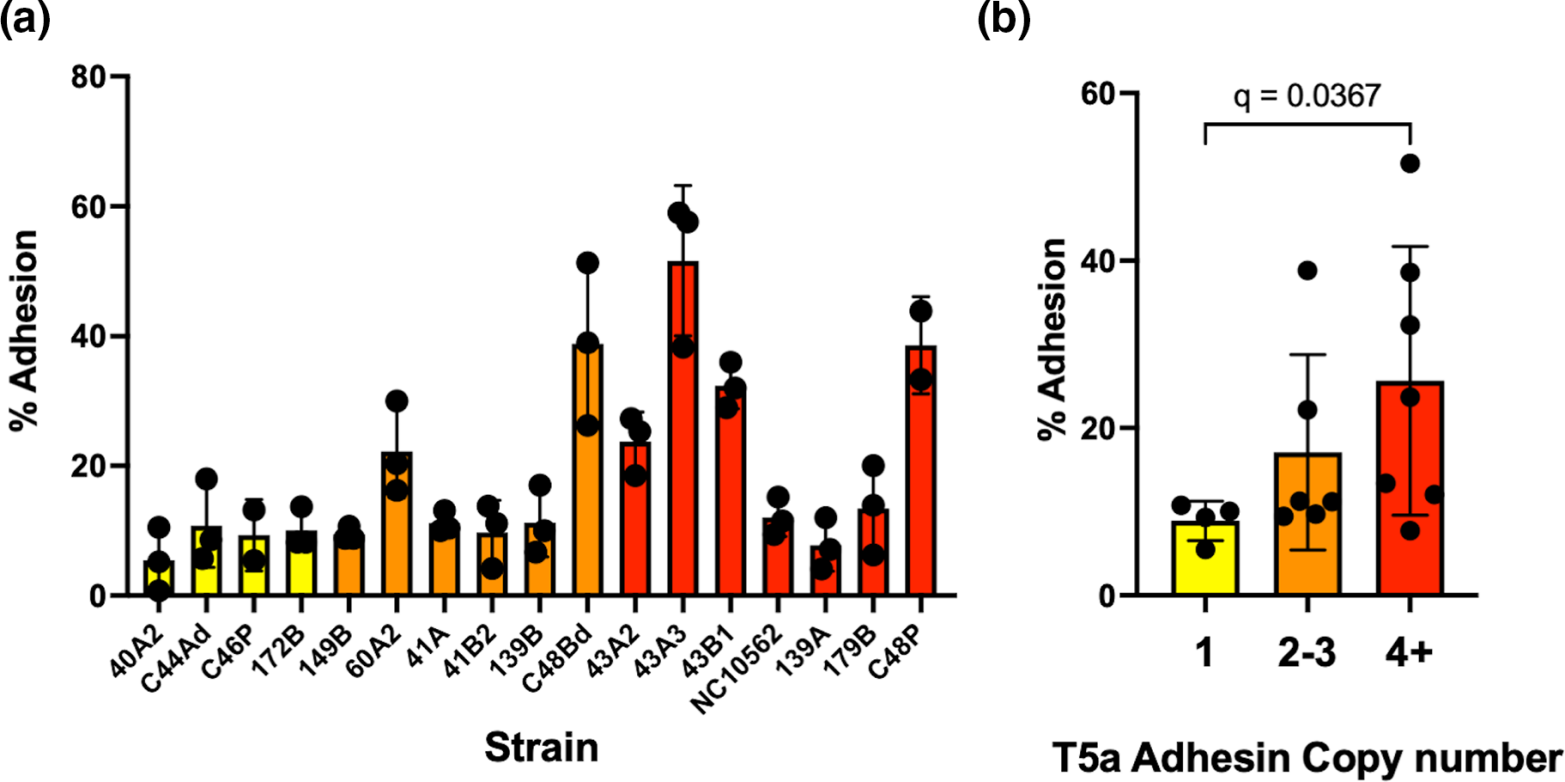
Analysis of adhesion to H376 oral keratinocytes by *F. nucleatum* subsp. *polymorphum* isolates. (**a**) Percentage adhesion to H376 oral keratinocytes by the indicated strains of *F. nucleatum* subsp. *polymorphum,* coloured by TVa adhesin copy number in the genome (yellow=1, orange=2–3, red=4 or more). (**b**) Average adhesion of isolates grouped by number of encoded TVa adhesins in the genome. The q value refers to Kruskall–Wallis test with Dunn’s multiple comparisons test.

We assessed adhesion to oral keratinocytes using the H376 cell line. We selected 17 *F*. *nucleatum* subsp. *polymorphum* isolates based on variations in copy number of TVa adhesins and copy number variation of *fadA*-related genes (*fadA2* and *fadA3*). Adhesion levels ranged from 5.5 % (40A2) to 51 % (43A3) (Fig. 7a). In general, strains with a single copy of a TVa adhesin gene formed fewer attachments to H376 cells (10.65 % adhesion) compared to strains with four or more adhesins (25.6 % adhesion) (Fig. 7b). However, not all high copy number isolates were strongly adherent. In particular 139A and 179B possessed five and seven TVa adhesin encoding genes respectively and exhibited adhesion patterns similar to low copy number isolates. Copy number of *fadA-*related genes did not influence adhesion to this cell line.

## Discussion

Multiple studies have described increased abundance of *F. nucleatum* in CRC tissues [8,61]. As an oral commensal, it is perhaps not surprising that recent studies have associated this bacterium with oral cancers and oral potentially malignant disorders such as OLK [17,19]. Despite the possible importance of this organism to oral health, there is a lack of information on the oral distribution of the *F. nucleatum* subspecies, their population structure and their potential role in malignant progression. Recent ground-breaking studies have shown that *F. nucleatum* is an important component of the intra-tumoral microbiome of OSCC where it exists in specific micro-niches where it can modulate local gene expression and immune cell infiltration and enhance keratinocyte motility [21].

In the current study, *F. nucleatum* subsp. *polymorphum* is the most commonly recovered *Fusobacterium* species from healthy and OLK sites, representing 75 % of isolates from OLK (Fig. 1a). The remaining organisms recovered included *F. nucleatum* subsp. *animalis*, *F. periodonticum* and a single isolate of *F. nucleatum* subsp. *vincentii*. This distribution is similar to that described by Krieger *et al*. who recently reported that in healthy dental plaque, *F. nucleatum* subsp. *polymorphum* is the dominant subspecies, followed by subspecies *animalis* and *vincentii* [5]. Eren *et al*. also reported that *F. nucleatum* subsp. *polymorphum*, *animalis* and *vincentii* make up the majority of *F. nucleatum* sequences recovered from mucosal surfaces [62]. Both studies indicate that * F. nucleatum* subsp. *nucleatum* is a minor component of the oral microbiome. Unlike Krieger *et al*. who found increased * F. nucleatum* subsp. *animalis* in oral abscesses, we did not observe increased subsp. *animalis* on OLK and found a relatively low incidence of subsp. *vincentii* and no subsp. *nucleatum* [62]. However, the biology of OLK is very different from an inflamed abscess so we cannot assume that *F. nucleatum* subsp. *animalis* will also be enriched on OLK. Although the selective media employed (JVN FAA) has been shown to support growth of *F. nucleatum*, we cannot exclude the possibility that these conditions introduce bias for particular subspecies [38]. Loss of viability during transport or general low abundance in the sample may also have precluded identification of some subspecies, particularly *F. nucleatum* subsp. *nucleatum*. It should also be noted that sequence-based studies may also report the incidence of unculturable strains that cannot be isolated *in vitro*.

Based on the high prevalence of *F. nucleatum* subsp. *polymorphum* reported here, we proceeded to investigate the population and genome structure of this subspecies. There appears to be no obvious association between *F. nucleatum* subsp. *polymorphum* genotype and OLK (Fig. S2). Isolates recovered from healthy mucosa, plaque and different OLK severities can be found with in all of the major clades in our phylogenetic analysis suggesting that *F. nucleatum* subsp. *polymorphum* strains found at sites of disease are not different from strains found on healthy surfaces (Fig. S2). Geography seems to be a clearer differential between isolates, with the majority of Korean isolates (10/15) found in Clade B and in PCA Group 2. Isolates which originate from the same patient, whether from OLK or a healthy site, tend to cluster together on the tree indicating a high degree of relatedness (Figs 1c and S2). This suggests that each individual may possess a unique strain of *F. nucleatum* subsp. *polymorphum* which may colonize multiple oral niches.

Analysis of the pangenome of *F. nucleatum* subsp. *polymorphum* revealed a relatively small core genome of 1604 genes (present in 95 % of isolates, Fig. 2). The large accessory genome points towards an open pangenome, which may be the result of many HGT and recombination events in the population. Annotation of the accessory genome with EggNOG showed a large number of phage and transposon encoded genes as well as the presence of a significant number of sequences with greater homology to *Bacilli* and *Clostridia* rather than *Fusobacteria* (Table S4). This high level of genetic exchange with *Firmicutes* has been described previously. Mira *et al*. highlighted the composite nature of the *F. nucleatum* genome and suggested that the bacterium’s evolution may have been facilitated by acquisition of amino acid fermentation pathways from *Clostridia* [33]. It is noteworthy that the ornithine catabolism operon identified in the majority of PCA group 2 isolates is also found widely in *Clostridiaceae* [62]

Perhaps the most interesting facet of the pangenome is the variation in copy number of adhesin encoding genes of the TVa autotransporter and FadA families (Fig. 4). The most common adhesin is the TVa adhesin Fap2, which is present as a single copy gene in most isolates, suggesting a critical role in the bacterium’s survival compared to the other Type Va adhesins [26,29]. In contrast, *radD,* which encodes a protein reported to play a role in co-aggregation with streptococci, is only present in 12 isolates, suggesting perhaps that this adhesin only imparts a selective advantage in specific hosts or niches [24]. Adhesin repertoire can even differ in genetically related strains from the same patient; for example, isolate 149A has additional copies of *cmpA* and *fadA3* compared to isolate 149B (Fig. 4). Adhesin repertoire may be influenced by location in the oral cavity and the nature of interspecies interactions in the local microbiome. For example, the plaque-associated isolate C48P possesses additional adhesins compared to the genetically related mucosal isolate C48Bd recovered from the same patient, which could facilitate interactions in the plaque microbiome. Hybrid Illumina/MinION assemblies for all isolates would be desirable to fully validate all of our findings.

The genomic locations of adhesin genes found in the core genome, including *fap2*, *fadA* and *fusolisin*, are conserved across the isolates (Fig. 5). The region of the genome between *fadA* and *fap2* appears to be a location exhibiting a large degree of variation in terms of TVa autotransporter complement. This variation in adhesion copy number across the isolates may be an indication of pathogenicity as we noted that isolates which possessed multiple TVa adhesins generally showed higher adhesion to H376 oral keratinocytes compared to those with a single TVa adhesin (Fig. 7b). This enhanced ability to adhere to epithelial cells means that these isolates may also be more likely to invade human epithelial cells and induce cell changes that have previously been noted, such as increased proliferation and increased cytokine production [31,63]. However, some isolates with multiple TVa adhesins exhibited relatively low adherence, suggesting expression levels or function may vary between isolates. Predicting virulence from genome sequence data in *Fusobacterium* species has previously proven difficult [64]. For example, Umana *et al*. demonstrated that *F. necrophorum* subsp. *funduliforme* showed better adherence to HCT116 cells than *F. nucleatum*, despite the fact that the former has far fewer TVa adhesins, but instead possess more TVc adhesins than *F. nucleatum* [65]. It is highly likely that factors other than the TVa adhesins contribute to variability in adhesion, and reverse genetic approaches will be required to elucidate the function of novel adhesins.

The variation in TVa adhesin repertoire in *F. nucleatum* subsp. *polymorphum* suggests that recombination and HGT play a major role in shaping virulence potential. Previous work by Tran *et al*. has suggested that HGT may be responsible for *fap2* and *radD* variation [66]. Here, we present analysis of TVa adhesin gene sequences that reveals highly mosaic gene structures shaped by recombination between different lineages of subspecies *polymorphum* and other *F. nucleatum* subspecies. The core adhesin *fap2* exhibits evidence of extensive recombination including genetic lineages from all *F. nucleatum* subspecies (Fig. 6a). Analysis of *cmpA*, *radD, fusolisin* and the *fvcE* autotransporter indicate a similar level of recombination and present strong evidence for HGT between different subspecies, including subspecies *polymorphum*, *nucleatum*, *animalis* and *vincentii* (Figs S18–S27). Analysis of several housekeeping genes did not reveal significant levels of recombination at these loci, suggesting that autotransporter sites may be hotspots for recombination (Figs S8–S17). This may be related to the large size of these genes and potential redundancy in TVa adhesin function. It is interesting to note that *F. nucleatum* subsp. *animalis* is the subspecies with which *F. nucleatum* subsp. *polymorphum* shares the most mosaicism (Figs S20–S27) and was also the second most common subspecies isolated by our direct culture method. The close proximity of these subspecies in the oral cavity may facilitate increased exchange of genetic information between them. These analyses also indicate that genes from *F. nucleatum* subsp. *polymorphum* tend to form distinct lineages compared to genes from subspecies *nucleatum*, *animalis* and *vincentii,* which more often tended to cluster together as a single lineage. For example, the *fadA* gene sequences from subspecies *nucleatum*, *animalis* and *vincentii* formed a single distinct lineage separate from subspecies *polymorphum* (Fig. S28). This may indicate that there are fewer barriers (e.g. restriction modification systems) limiting HGT between subspecies *nucleatum*, *animalis* and *vincentii* compared to subspecies *polymorphum*.

In conclusion, we have found that *F. nucleatum* subsp. *polymorphum* is the most commonly isolated *F. nucleatum* subspecies recovered from healthy mucosa and potentially malignant OLK. The open nature of the pangenome and prevalence of HGT results in a population with a highly variable complement of potential virulence genes. It is likely that local host factors (e.g. host glycosylation patterns, local microbiome) drive selection of adhesin repertoires that facilitate oral colonization. Recent advances in the molecular genetics of *F. nucleatum* have allowed investigators to begin the process of teasing out the relative roles of this large body of adhesins in bacterial–host interactions [31,67]. The preponderance of *F. nucleatum* subsp. *polymorphum* on oral sites suggests that extension of these tools to this subspecies would greatly assist in dissecting the role of this bacterium in the development of oral malignancies.

## supplementary material

10.1099/mgen.0.001217 Uncited Fig. S1.

10.1099/mgen.0.001217 Uncited Table S1.
