# Supplementary material for: Fusobacterium nucleatum subsp. polymorphum recovered from malignant and potentially malignant oral disease exhibit heterogeneity in adhesion phenotypes and adhesin gene copy number, shaped by inter-subspecies horizontal gene transfer and recombination-derived mosaicism
Source: Microb Genom. 2024 Mar 26;10(3):001217. doi: 10.1099/mgen.0.001217 (PMC10995627; doi:10.1099/mgen.0.001217)
Supplement: Uncited Fig. S1. [file mgen-10-01217-s001.pdf]

Figure S1

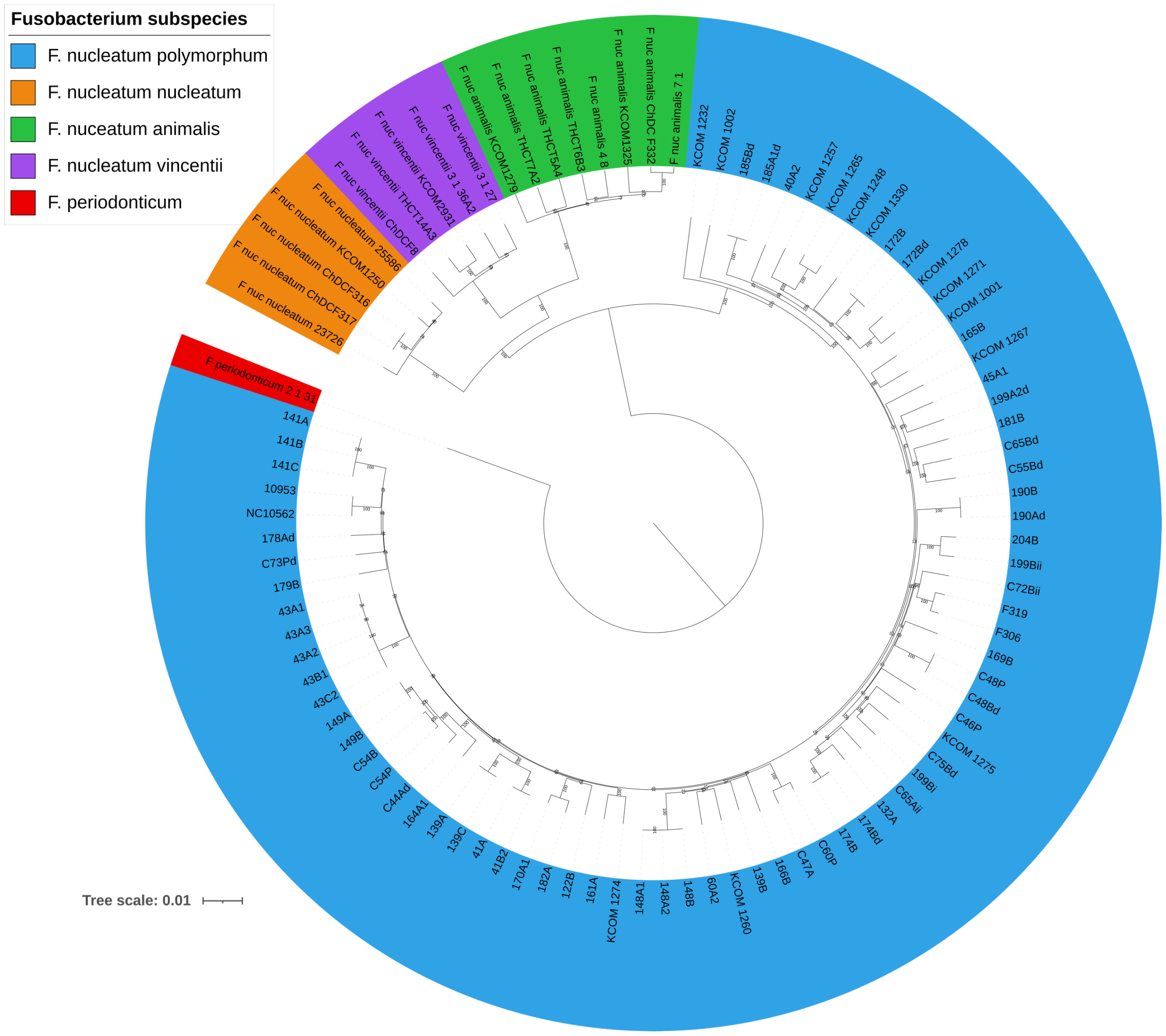

Figure S1. Neighbour-joining phylogenetic tree generated from an alignment of the core genome (n=173 genes) identified in the indicated *Fusobacterium* genomes using Panaroo. Core genes from *F. periodonticum* (red) *F. nucleatum* subspecies *animalis* (green), *F. nucleatum* subspecies *nucleatum* (orange) and *F. nucleatum* subspecies *vincentii* (purple) are clearly separated from the *F. nucleatum* subspecies *polymorphum* isolates collected here (blue). Bootstrap values for all branches separating different subspecies =100.

Figure S2

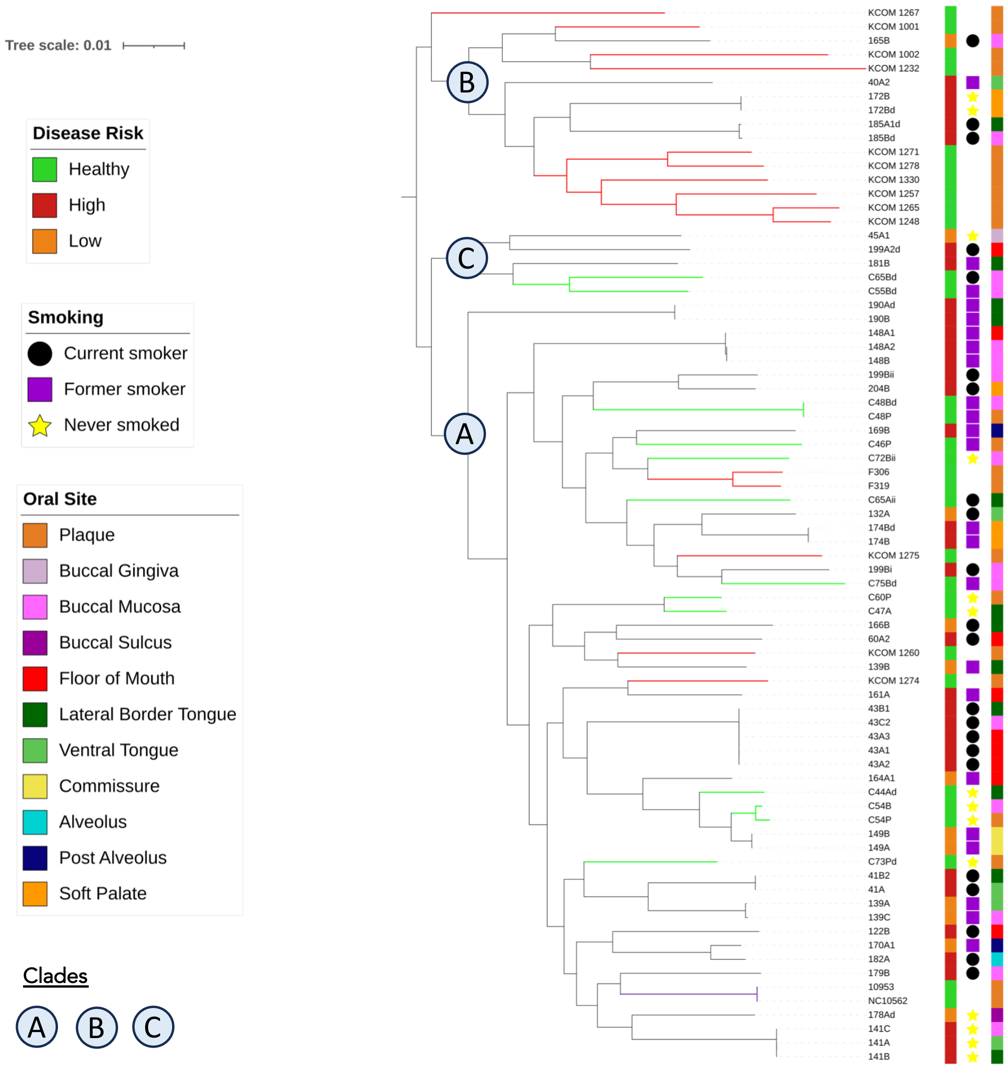

Figure S2. Phylogenetic tree showing the relationship between *Fusobacterium nucleatum* subsp. *polymorphum* genotype and host factors. Tree is generated from the core genome alignment produced in Panaroo. The three main clades are indicated (A, B, C). Disease risk refers to samples from healthy volunteers, patient samples from sites of low risk of malignant change (mild dysplasia) and high risk (moderate and severe dysplasia). Smoking status (current, former never) and site of sampling are also indicated.

Figure S3

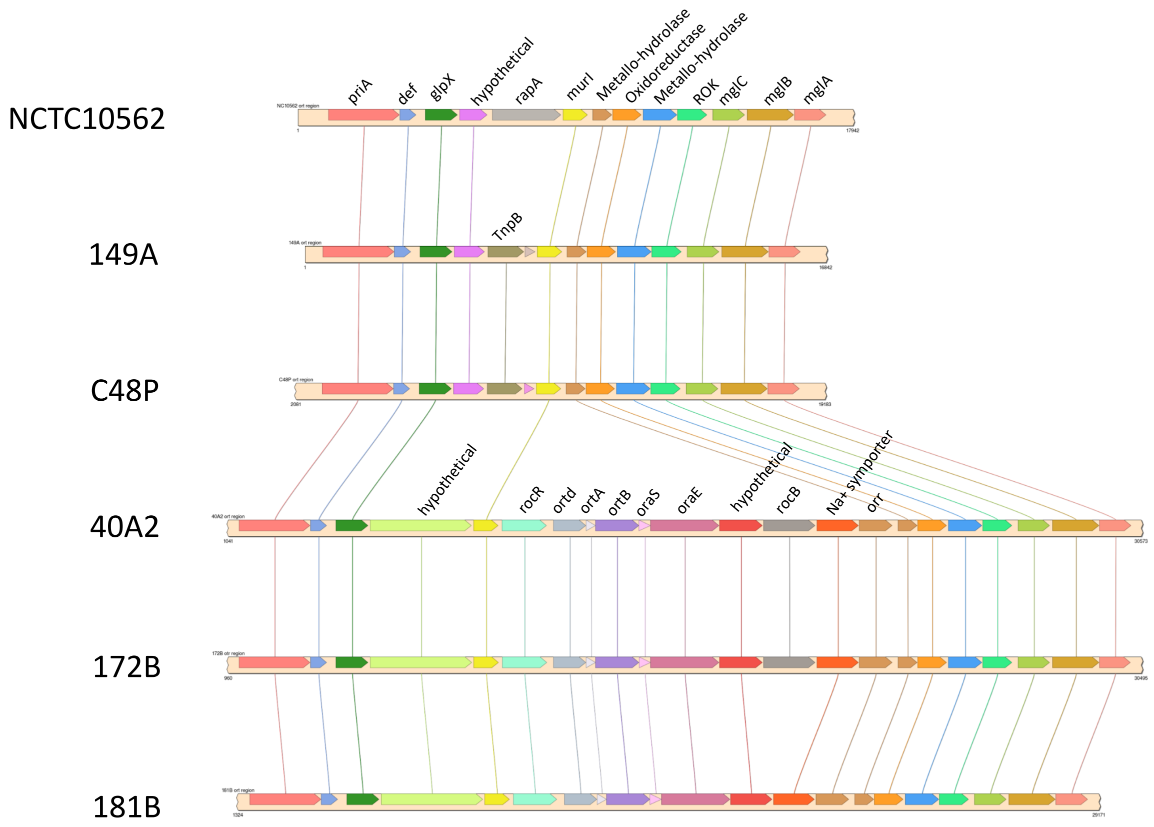

Figure S3. Structure of the ornithine catabolism operon identified in PCA group 2 isolates. Genome wide association analysis identified a high prevalence of an operon encoding genes required for ornithine catabolism present in 15/22 PCA group 2 isolates (including 40A2, 172B and 181B shown here) and absent in PCA group 1 isolates (including NCTC10562, 149A and C48P shown here). Graphics were generated using Simple Synteny https://www.dveltri.com/simplesynteny/.

Figure S4

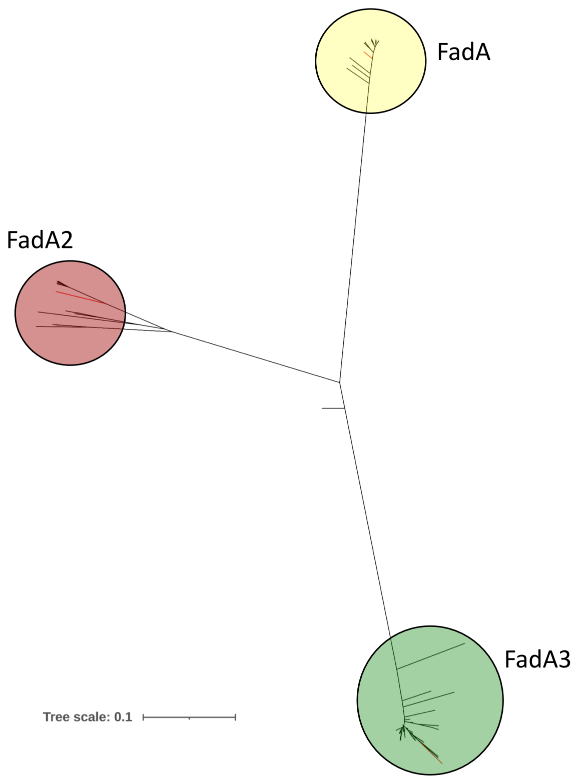

Figure S4. Phylogenetic tree showing the similarity of FadA, FadA2 and FadA3 proteins in *F. nucleatum* subsp. *polymorphum*. Alignment was generated in MAFFT and Maximum likelihood phylogenetic tree visualised in FigTree. Genes from *F. nucleatum* subsp. *polymorphum* 23726 were included for reference (red branches).

Figure S5

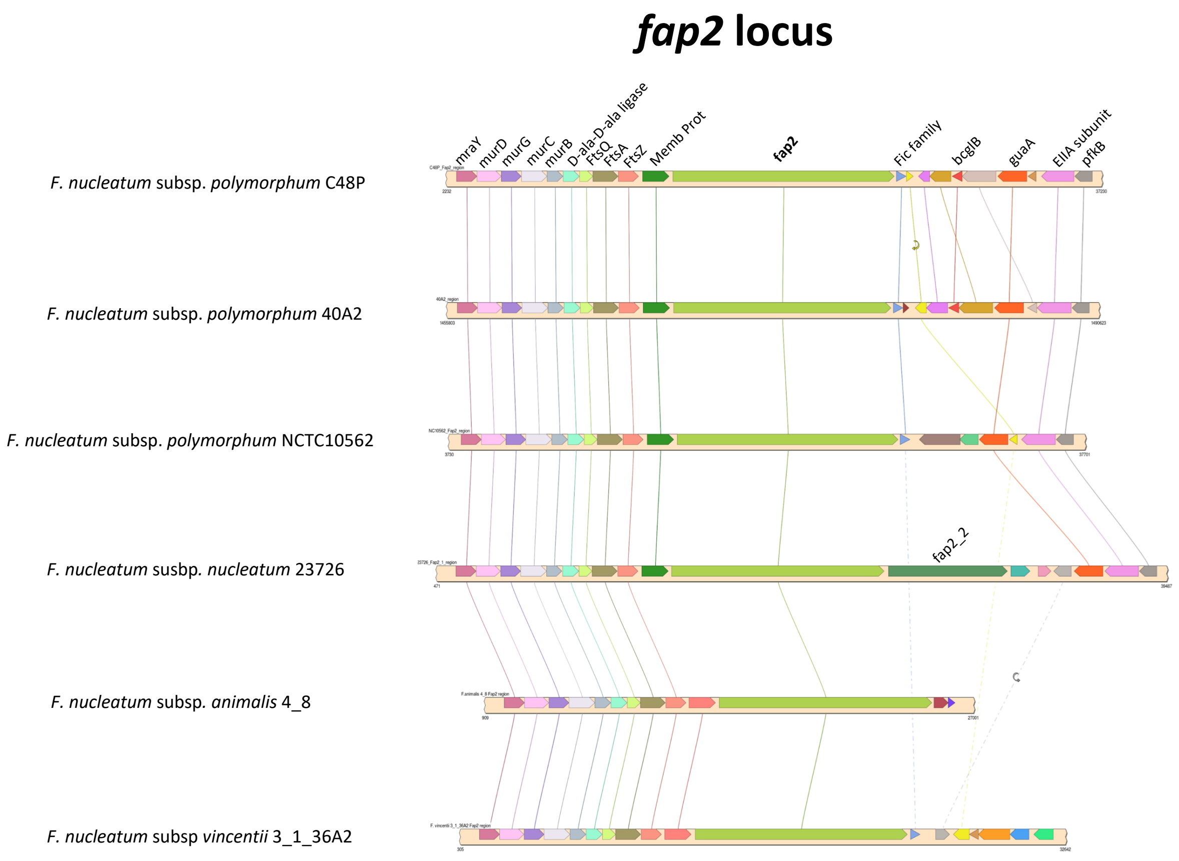

Figure S5. Diagram of the *fap2* locus in *F. nucleatum*. Orthologous genes in the indicated strains are connected by lines. Curved arrows indicate gene inversions. Gene annotations were determined in Prokka. Graphics were generated using Simple Synteny https://www.dveltri.com/simplesynteny/

Figure S6

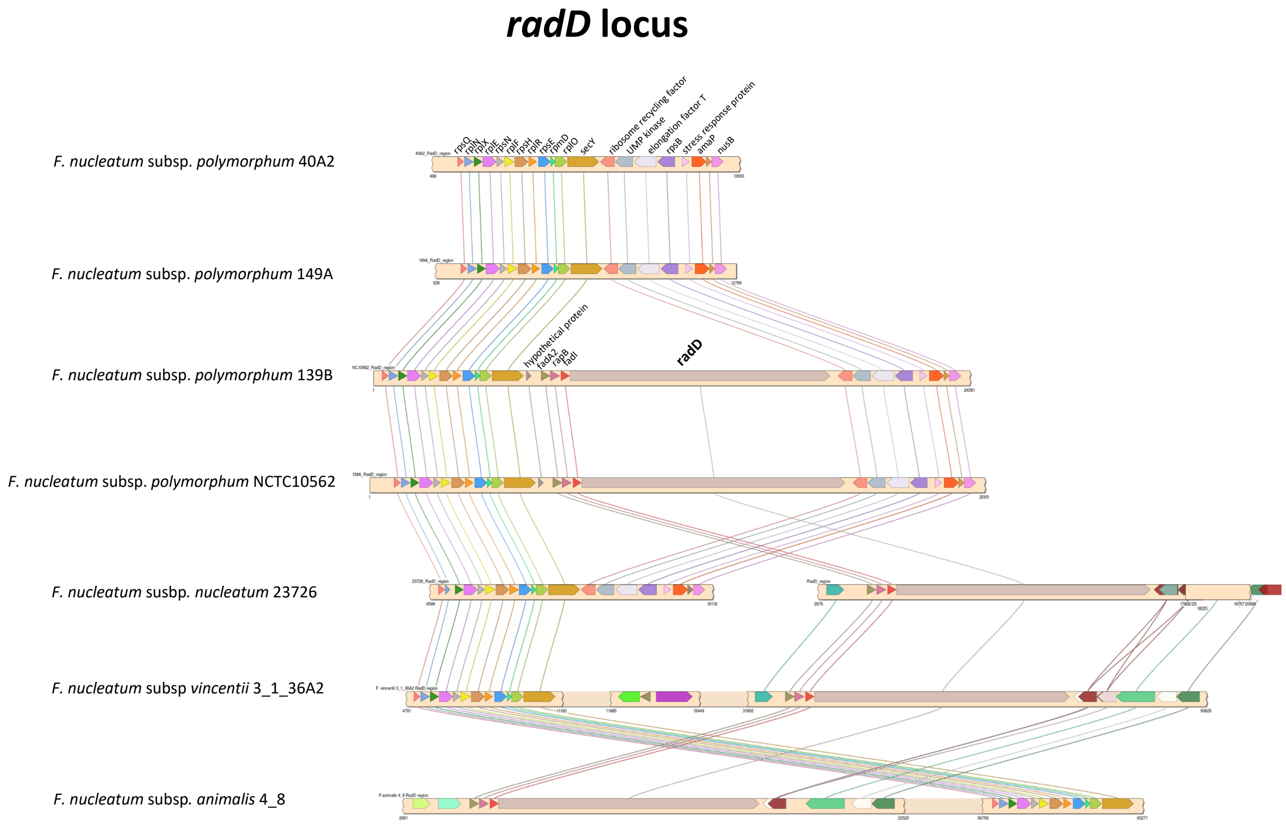

Figure S6. Diagram of the *radD* locus in *F. nucleatum*. Orthologous genes in the indicated strains are connected by lines. Curved arrows indicate gene inversions. Gene annotations were determined in Prokka. Graphics were generated using Simple Synteny https://www.dveltri.com/simplesynteny/

Figure S7

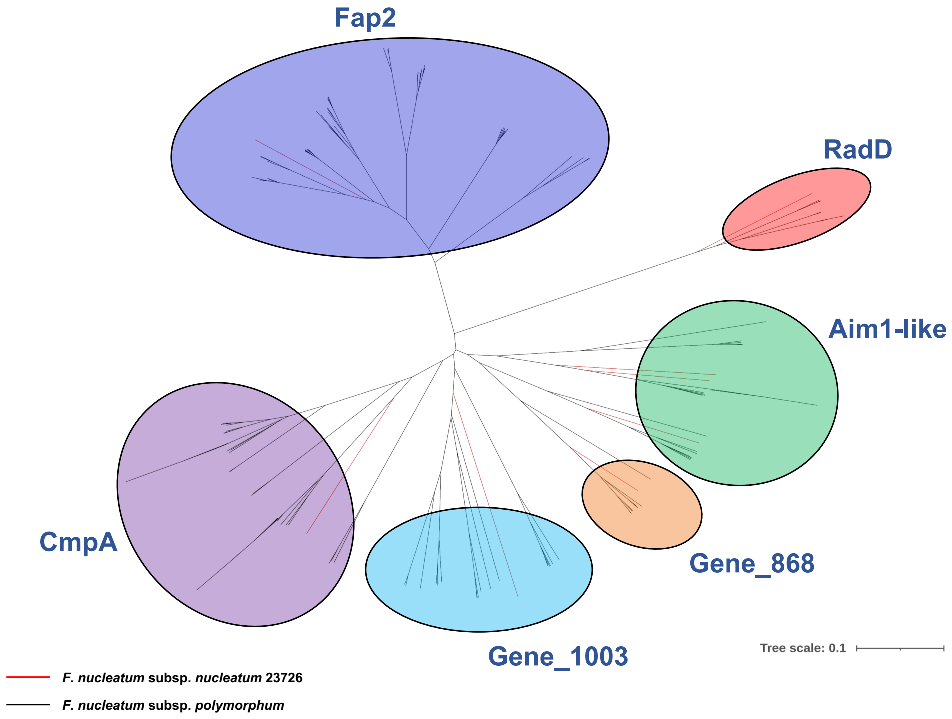

Figure S7. Phylogenetic tree showing the similarity of Type Va autotransporter proteins in *F. nucleatum* subsp. *polymorphum*. Autotransporters were identified by HMMER searches of the *F. nucleatum* subsp. *polymorphum* pangenome. Alignment was generated in MAFFT and Maximum likelihood phylogenetic tree visualised in FigTree. Genes from *F. nucleatum* subsp. *polymorphum* 23726 were included for reference (red branches). The Aim1-like cluster includes Aim1, Gene_351 and Gene_665 from strain 23726.

Figure S8

***gyrB:* Recent recombination**

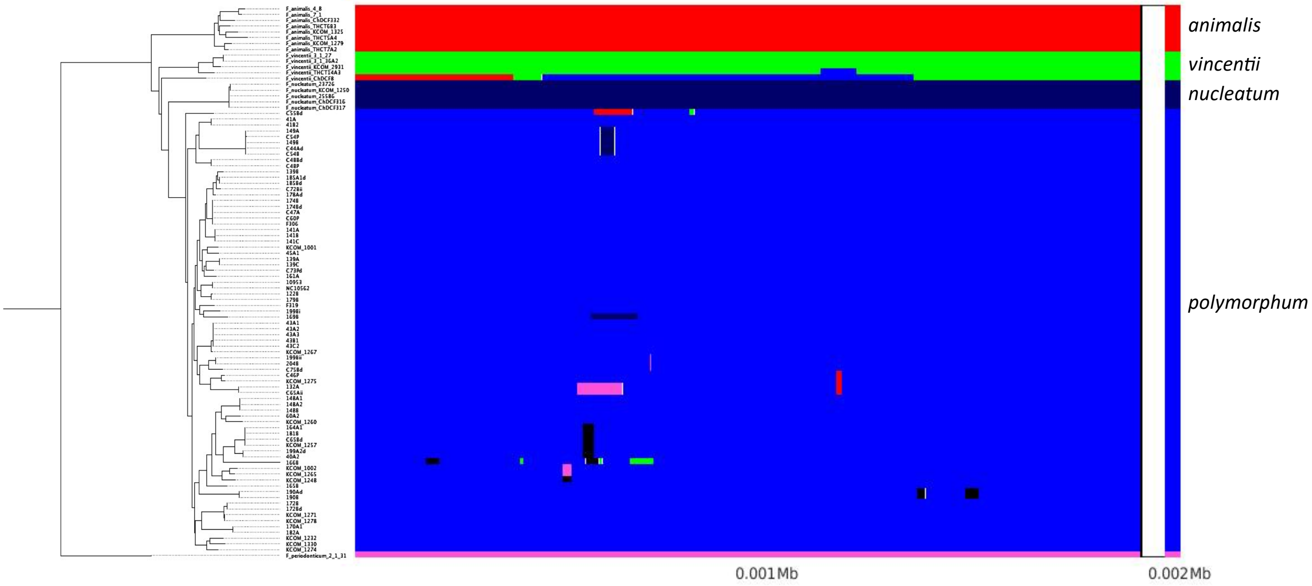

Figure S8. Analysis of recent recombination within the *gyrB* gene in *Fusobacterium* species using fastGEAR. Sequences of the *gyrB* gene from *F. periodonticum* and the indicated strains of *F. nucleatum* were aligned in MAFFT (v7.0) and subjected to analysis in fastGEAR to detect evidence of recombination. On the y-axis are the 95 sequences included in the sequence alignment ordered by position in a phylogenetic tree. The x-axis corresponds to the sequence positions of the respective genes. The coloured legend on the right side of the panel shows division of the strains into lineages indicated by different colours. Grey sections represent gaps in the sequence alignment.

Figure S9

***gyrB:* Ancestral recombination**

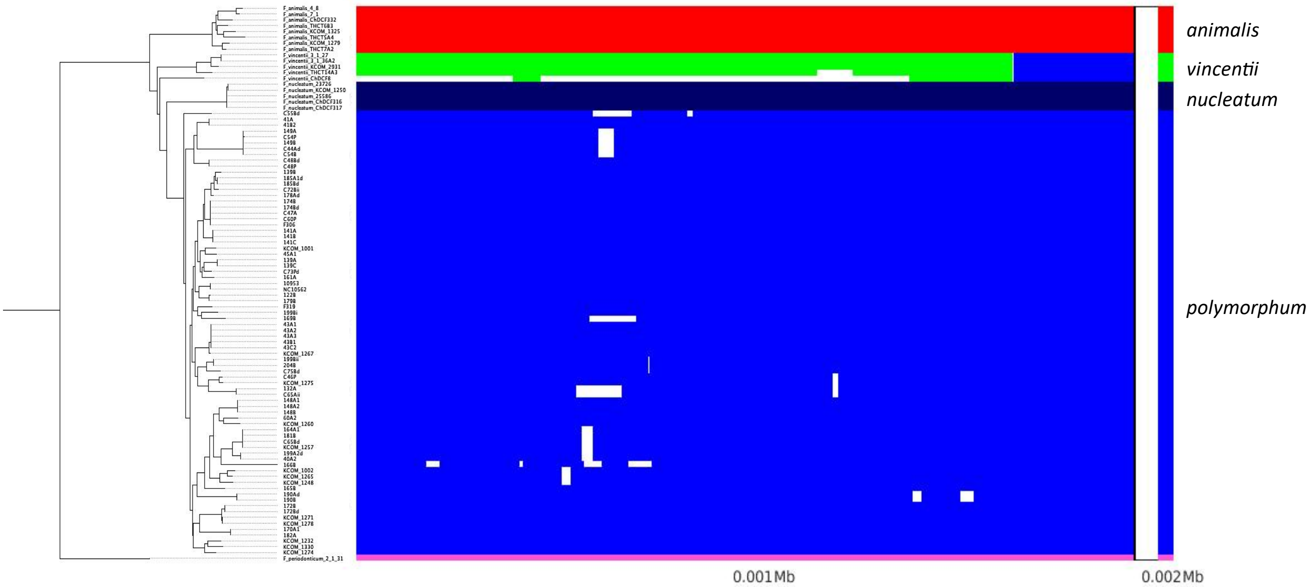

Figure S9. Analysis of ancestral recombination within the *gyrB* gene in *Fusobacterium* species using fastGEAR. Sequences of the *gyrB* gene from *F. periodonticum* and the indicated strains of *F. nucleatum* were aligned in MAFFT (v7.0) and subjected to analysis in fastGEAR to detect evidence of recombination. On the y-axis are the 95 sequences included in the sequence alignment ordered by position in a phylogenetic tree. The x-axis corresponds to the sequence positions of the respective genes. The coloured legend on the right side of the panel shows division of the strains into lineages indicated by different colours. White sections represent recent recombination shown in Fig S9.

Figure S10

***recA:* Recent recombination**

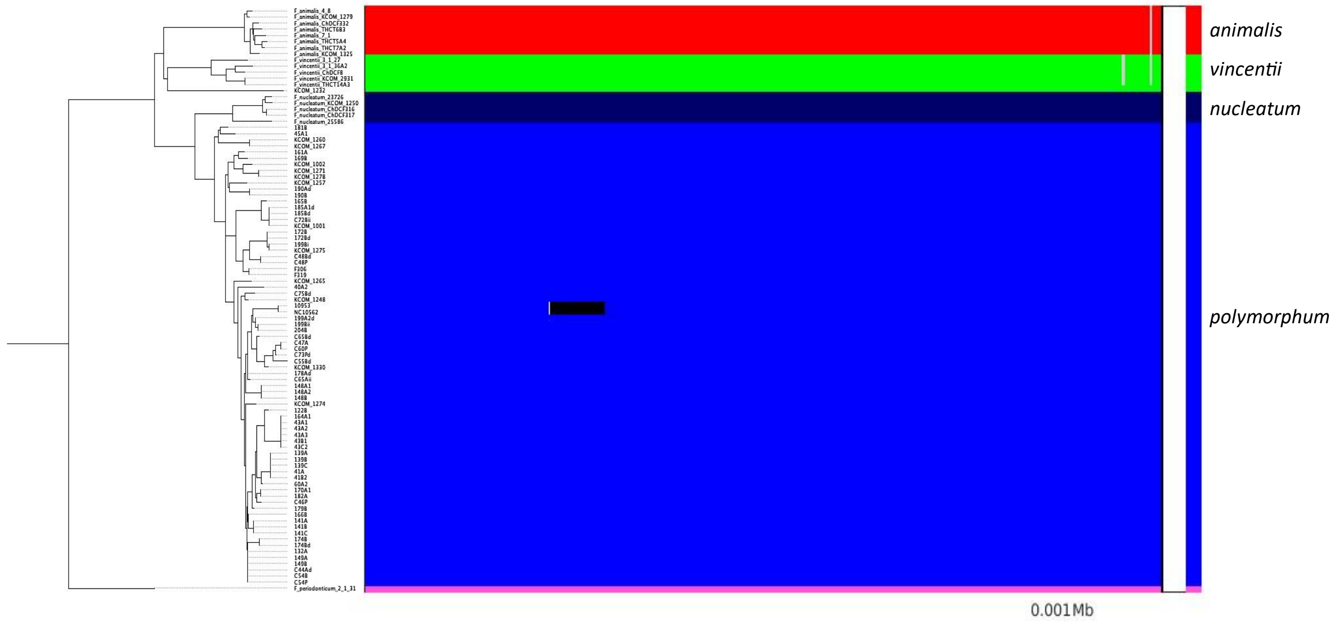

Figure S10. Analysis of recent recombination within the *recA* gene in *Fusobacterium* species using fastGEAR. Sequences of the *recA* gene from *F. periodonticum* and the indicated strains of *F. nucleatum* were aligned in MAFFT (v7.0) and subjected to analysis in fastGEAR to detect evidence of recombination. On the y-axis are the 95 sequences included in the sequence alignment ordered by position in a phylogenetic tree. The x-axis corresponds to the sequence positions of the respective genes. The coloured legend on the right side of the panel shows division of the strains into lineages indicated by different colours.

Figure S11

recA: Ancestral recombination

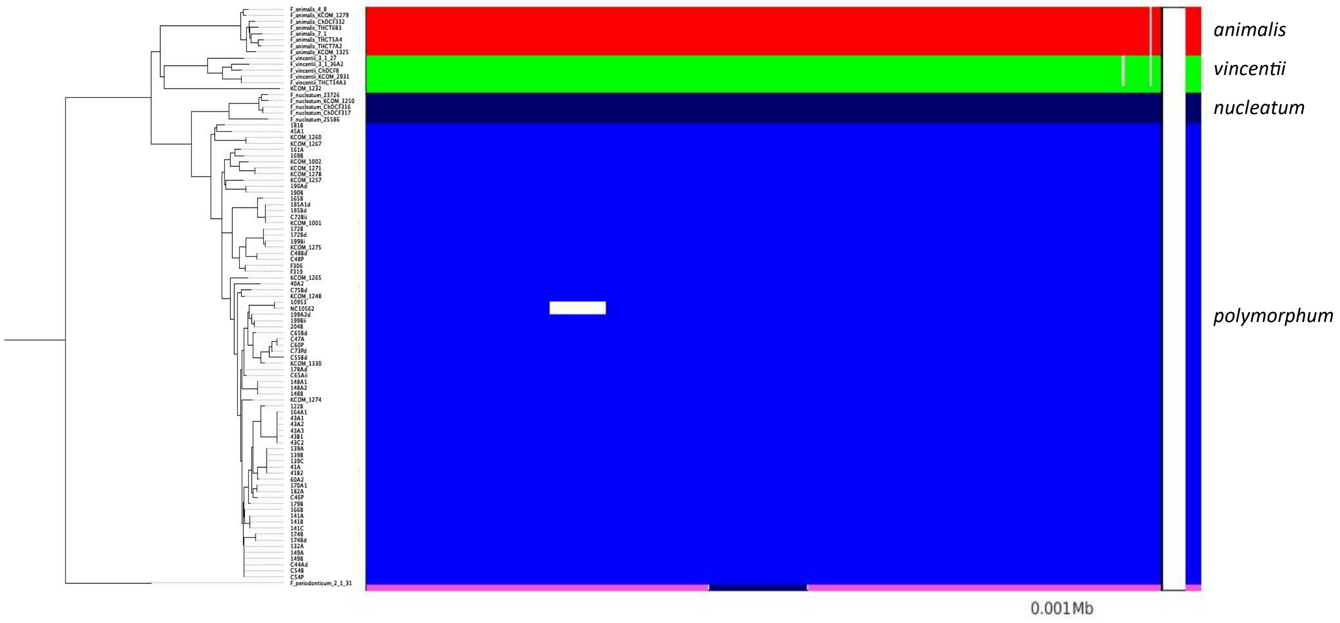

Figure S11. Analysis of ancestral recombination within the *recA* gene in *Fusobacterium* species using fastGEAR. Sequences of the *recA* gene from *F. periodonticum* and the indicated strains of *F. nucleatum* were aligned in MAFFT (v7.0) and subjected to analysis in fastGEAR to detect evidence of recombination. On the y-axis are the 95 sequences included in the sequence alignment ordered by position in a phylogenetic tree. The x-axis corresponds to the sequence positions of the respective genes. The coloured legend on the right side of the panel shows division of the strains into lineages indicated by different colours. White sections represent recent recombination shown in Fig S11.

Figure S12

***ruvB:* Recent recombination**

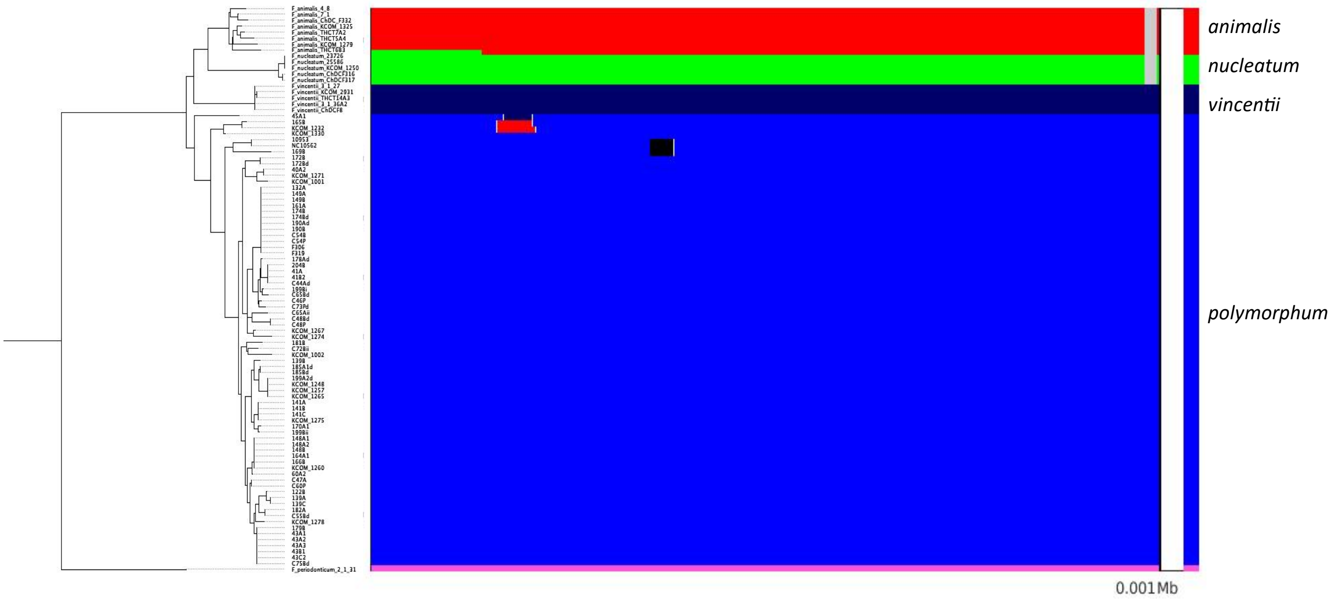

Figure S12. Analysis of recent recombination within the *ruvB* gene in *Fusobacterium* species using fastGEAR. Sequences of the *ruvB* gene from *F. periodonticum* and the indicated strains of *F. nucleatum* were aligned in MAFFT (v7.0) and subjected to analysis in fastGEAR to detect evidence of recombination. On the y-axis are the 95 sequences included in the sequence alignment ordered by position in a phylogenetic tree. The x-axis corresponds to the sequence positions of the respective genes. The coloured legend on the right side of the panel shows division of the strains into lineages indicated by different colours.

Figure S13

***ruvB:* Ancestral recombination**

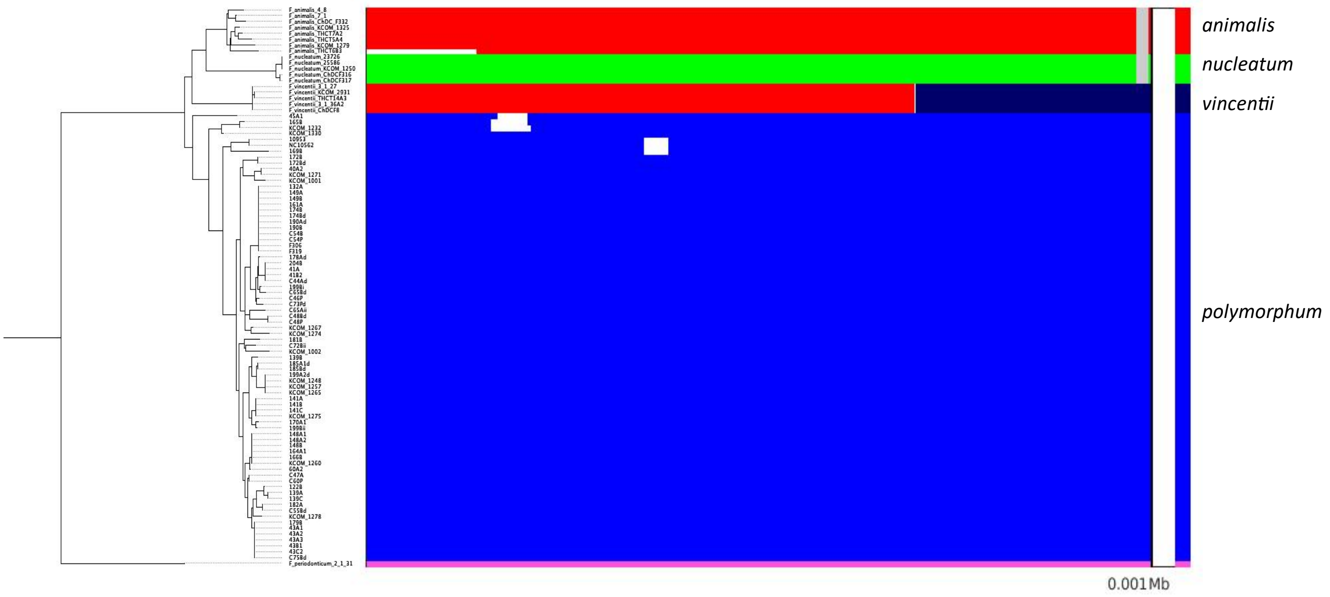

Figure S13. Analysis of ancestral recombination within the *ruvB* gene in *Fusobacterium* species using fastGEAR. Sequences of the *ruvB* gene from *F. periodonticum* and the indicated strains of *F. nucleatum* were aligned in MAFFT (v7.0) and subjected to analysis in fastGEAR to detect evidence of recombination. On the y-axis are the 95 sequences included in the sequence alignment ordered by position in a phylogenetic tree. The x-axis corresponds to the sequence positions of the respective genes. The coloured legend on the right side of the panel shows division of the strains into lineages indicated by different colours and named according to the subspecies represented within. White sections represent recent recombination shown in Fig S13.

Figure S14

***fstZ:* Recent recombination**

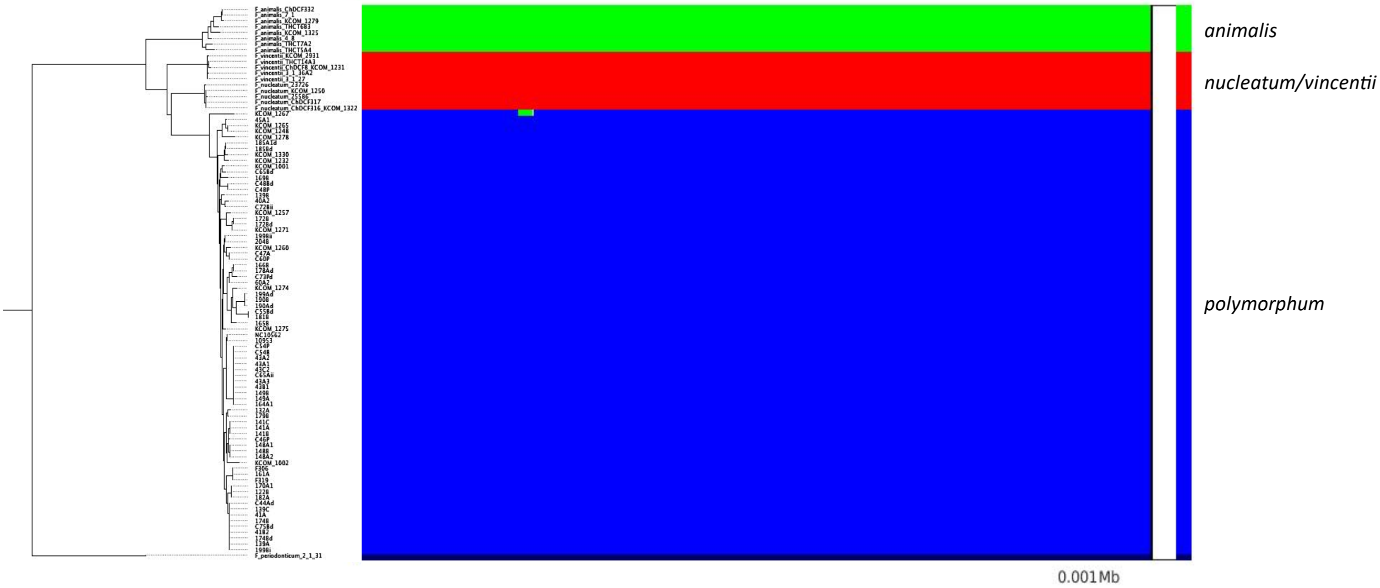

Figure S14. Analysis of recent recombination within the *ftsZ* gene in *Fusobacterium* species using fastGEAR. Sequences of the *ftsZ* gene from *F. periodonticum* and the indicated strains of *F. nucleatum* were aligned in MAFFT (v7.0) and subjected to analysis in fastGEAR to detect evidence of recombination. On the y-axis are the 95 sequences included in the sequence alignment ordered by position in a phylogenetic tree. The x-axis corresponds to the sequence positions of the respective genes. The coloured legend on the right side of the panel shows division of the strains into lineages indicated by different colours.

Figure S15

***ftsZ:* Ancestral recombination**

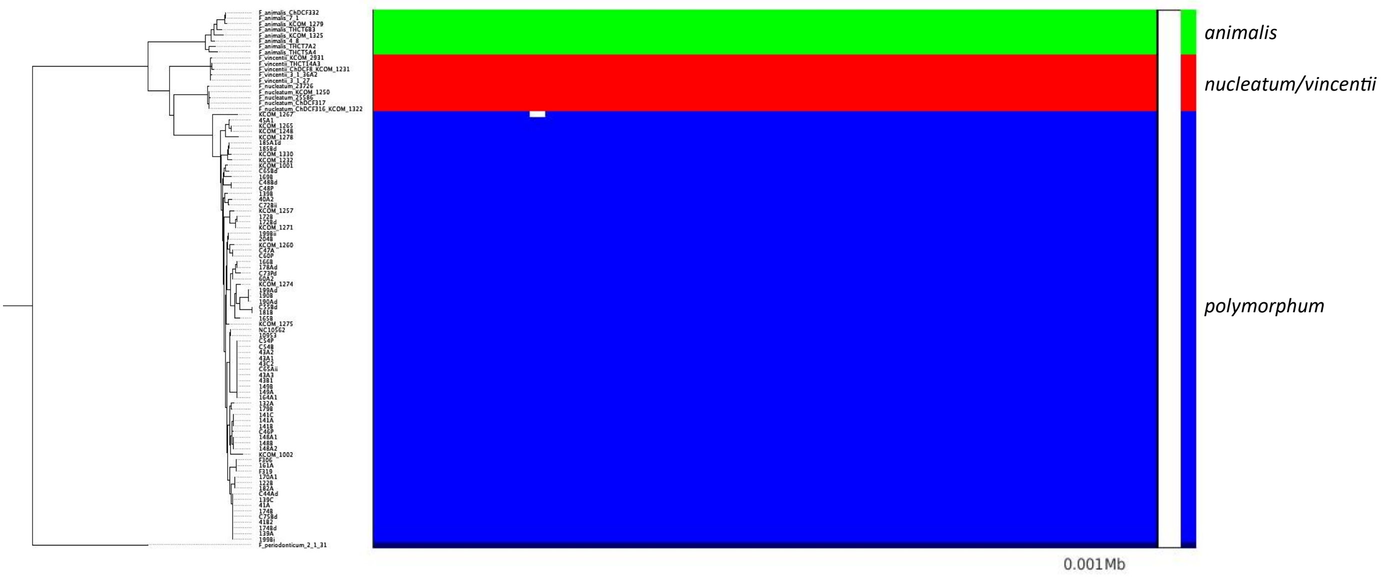

Figure S15. Analysis of ancestral recombination within the *fstZ* gene in *Fusobacterium* species using fastGEAR. Sequences of the *ftsZ* gene from *F. periodonticum* and the indicated strains of *F. nucleatum* were aligned in MAFFT (v7.0) and subjected to analysis in fastGEAR to detect evidence of recombination. On the y-axis are the 95 sequences included in the sequence alignment ordered by position in a phylogenetic tree. The x-axis corresponds to the sequence positions of the respective genes. The coloured legend on the right side of the panel shows division of the strains into lineages indicated by different colours and named according to the subspecies represented within. White sections represent recent recombination shown in Fig S15.

Figure S16

***rsmI:* Recent recombination**

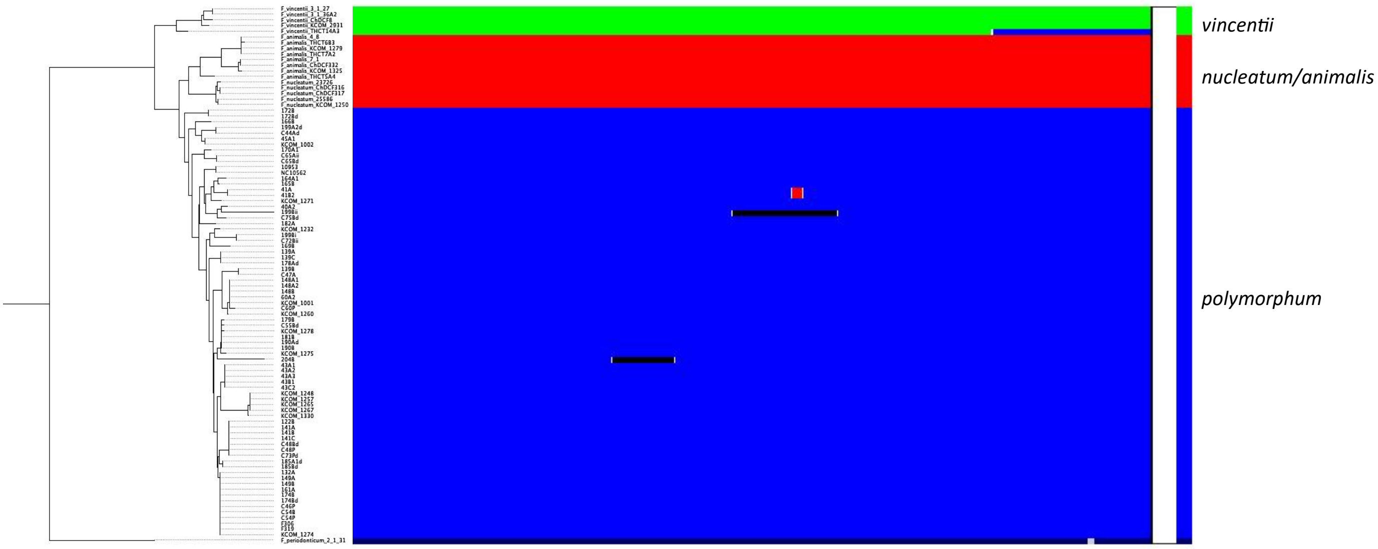

Figure S16. Analysis of recent recombination within the *rsmI* gene in *Fusobacterium* species using fastGEAR. Sequences of the *rsmI* gene from *F. periodonticum* and the indicated strains of *F. nucleatum* were aligned in MAFFT (v7.0) and subjected to analysis in fastGEAR to detect evidence of recombination. On the y-axis are the 95 sequences included in the sequence alignment ordered by position in a phylogenetic tree. The x-axis corresponds to the sequence positions of the respective genes. The coloured legend on the right side of the panel shows division of the strains into lineages indicated by different colours.

Figure S17

rsml: Ancestral recombination

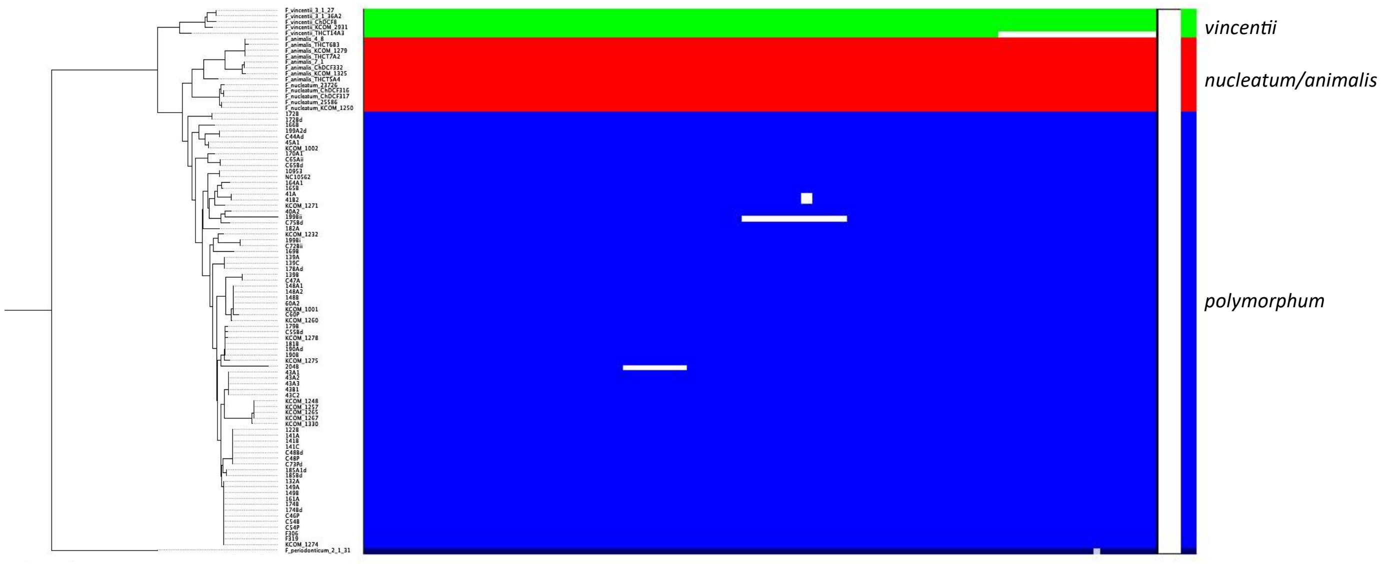

Figure S17. Analysis of ancestral recombination within the *rsml* gene in *Fusobacterium* species using fastGEAR. Sequences of the *rsml* gene from *F. periodonticum* and the indicated strains of *F. nucleatum* were aligned in MAFFT (v7.0) and subjected to analysis in fastGEAR to detect evidence of recombination. On the y-axis are the 95 sequences included in the sequence alignment ordered by position in a phylogenetic tree. The x-axis corresponds to the sequence positions of the respective genes. The coloured legend on the right side of the panel shows division of the strains into lineages indicated by different colours and named according to the subspecies represented within. White sections represent recent recombination shown in Fig S17.

Figure S18

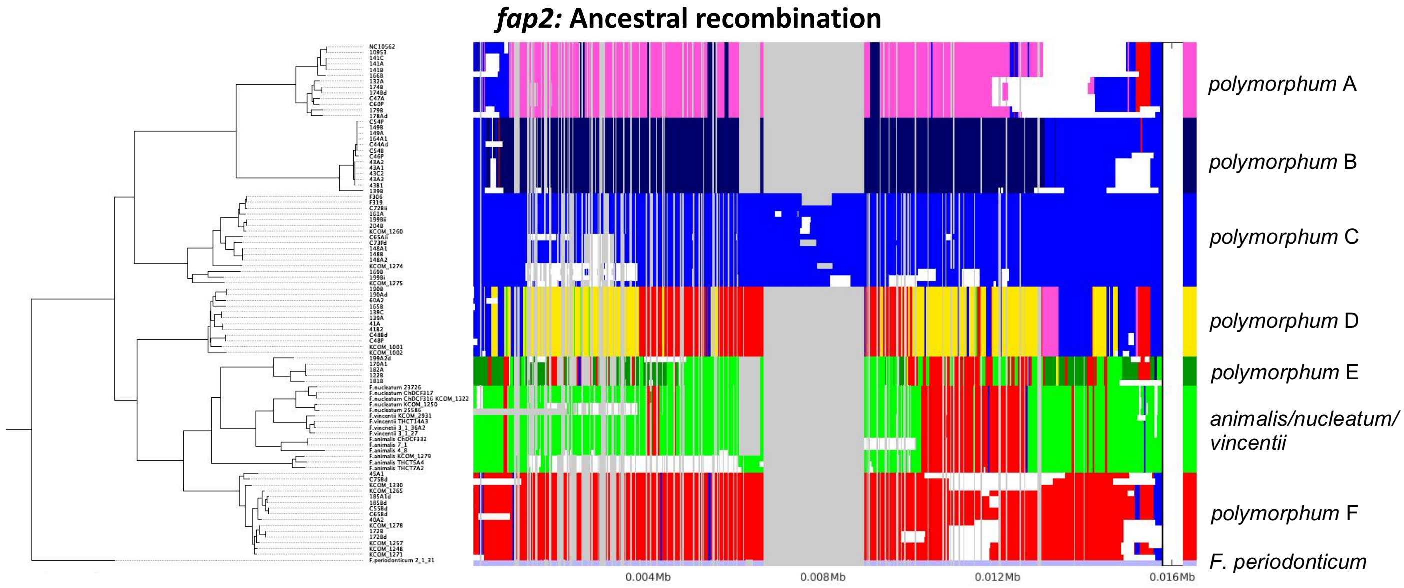

Figure S18. Analysis of recent recombination within the *fap2* gene in *Fusobacterium* species using fastGEAR. Sequences of the *fap2* gene from *F. periodonticum* and the indicated strains of *F. nucleatum* were aligned in MAFFT (v7.0) and subjected to analysis in fastGEAR to detect evidence of recombination. On the y-axis are the 90 sequences included in the sequence alignment ordered by position in a phylogenetic tree. The x-axis corresponds to the sequence positions of the respective genes. Regions in grey represent gaps in the alignment. The coloured legend on the right side of the panel shows division of the strains into lineages indicated by different colours.

Figure S19

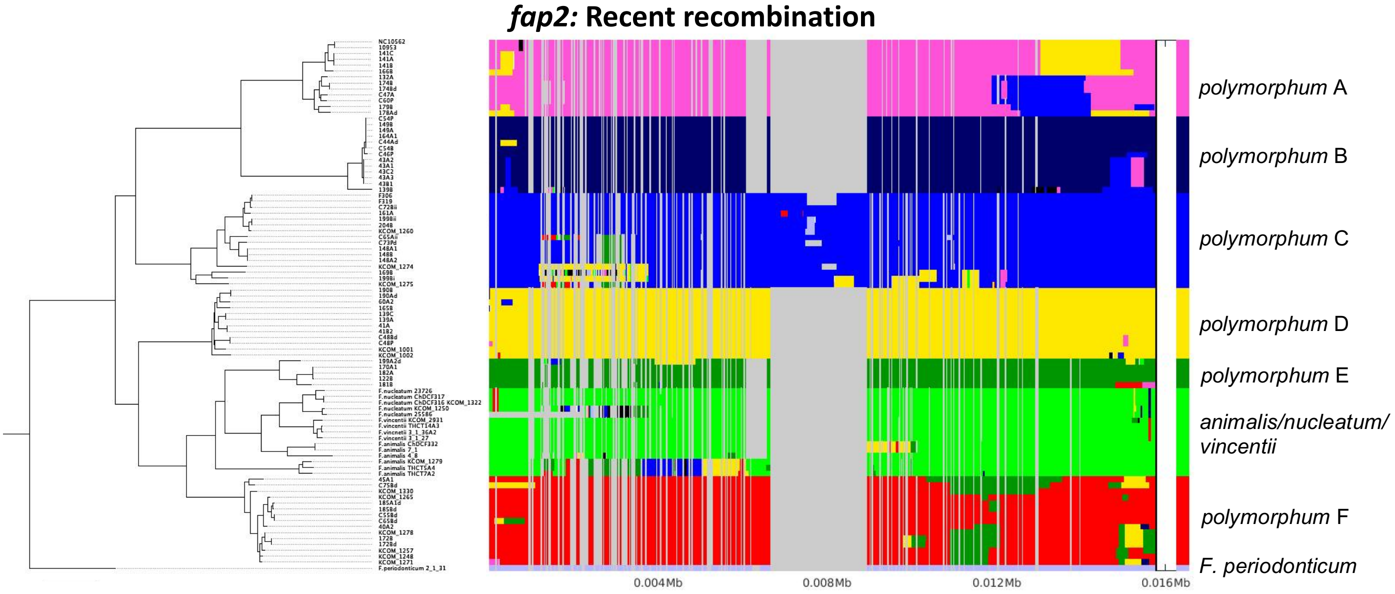

Figure S19. Analysis of ancestral recombination within the *fap2* gene in *Fusobacterium* species using fastGEAR. Sequences of the *fap2* gene from *F. periodonticum* and the indicated strains of *F. nucleatum* were aligned in MAFFT (v7.0) and subjected to analysis in fastGEAR to detect evidence of recombination. On the y-axis are the 90 sequences included in the sequence alignment ordered by position in a phylogenetic tree. The x-axis corresponds to the sequence positions of the respective genes. Regions in grey represent gaps in the alignment. The coloured legend on the right side of the panel shows division of the strains into lineages indicated by different colours and named according to the subspecies represented within. White sections represent recent recombination shown in Fig S19.

Figure S20

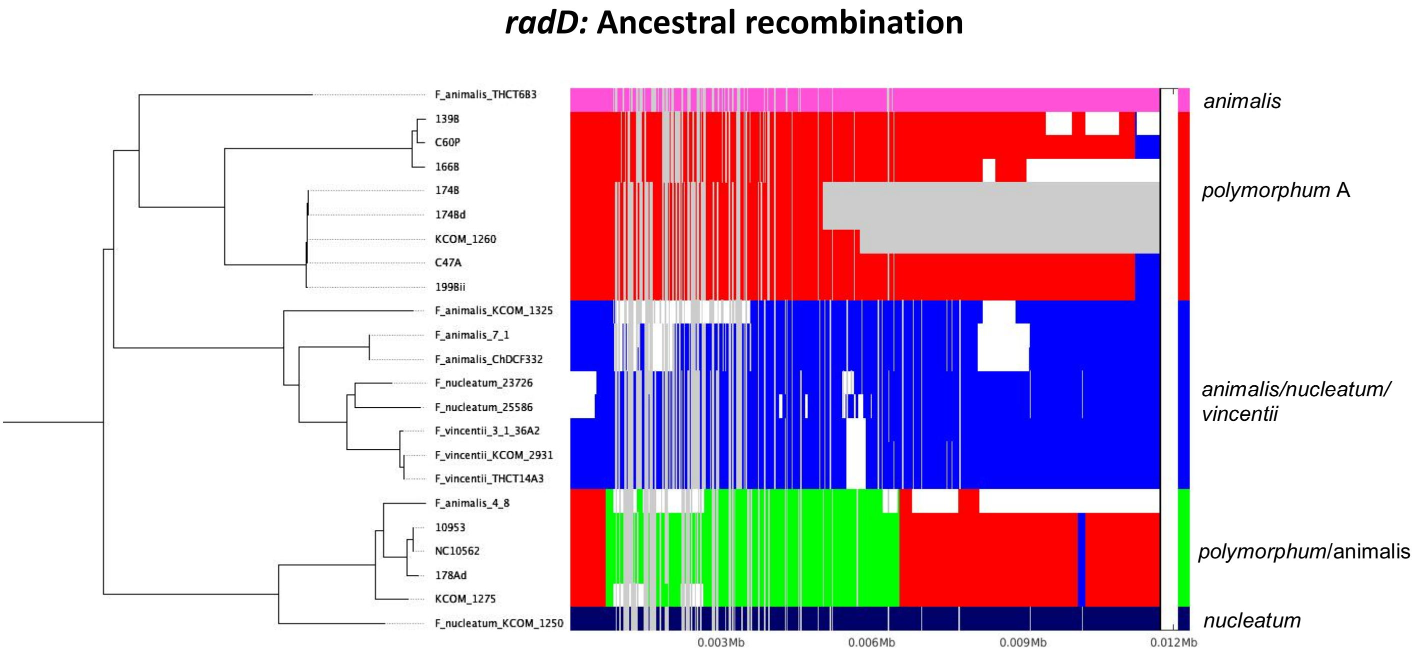

Figure S20. Analysis of recent recombination within the *radD* gene in *Fusobacterium* species using fastGEAR. Sequences of the *radD* gene from *F. periodonticum* and the indicated strains of *F. nucleatum* were aligned in MAFFT (v7.0) and subjected to analysis in fastGEAR to detect evidence of recombination. On the y-axis are the 23 sequences included in the sequence alignment ordered by position in a phylogenetic tree. The x-axis corresponds to the sequence positions of the respective genes. Regions in grey represent gaps in the alignment. The coloured legend on the right side of the panel shows division of the strains into lineages indicated by different colours.

Figure S21

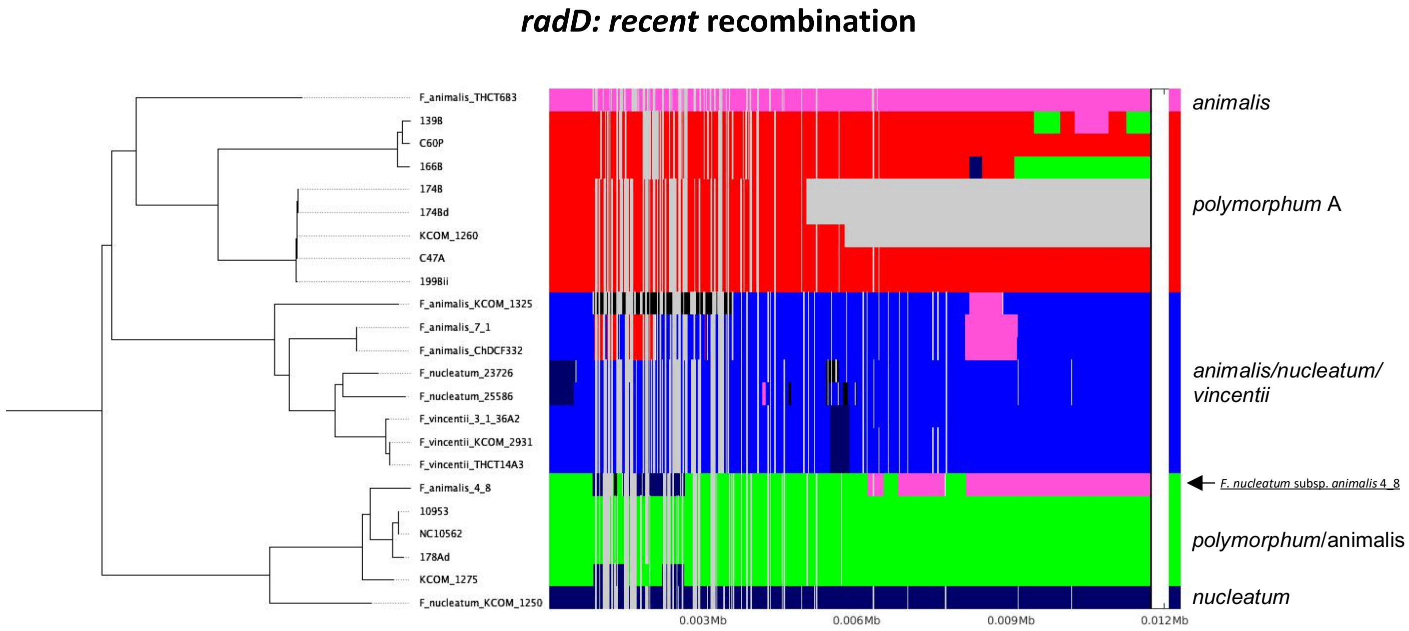

Figure S21. Analysis of ancestral recombination within the *radD* gene in *Fusobacterium* species using fastGEAR. Sequences of the *radD* gene from *F. periodonticum* and the indicated strains of *F. nucleatum* were aligned in MAFFT (v7.0) and subjected to analysis in fastGEAR to detect evidence of recombination. On the y-axis are the 23 sequences included in the sequence alignment ordered by position in a phylogenetic tree. The x-axis corresponds to the sequence positions of the respective genes. Regions in grey represent gaps in the alignment. The coloured legend on the right side of the panel shows division of the strains into lineages indicated by different colours. White sections represent recent recombination shown in Fig S21.

Figure S22

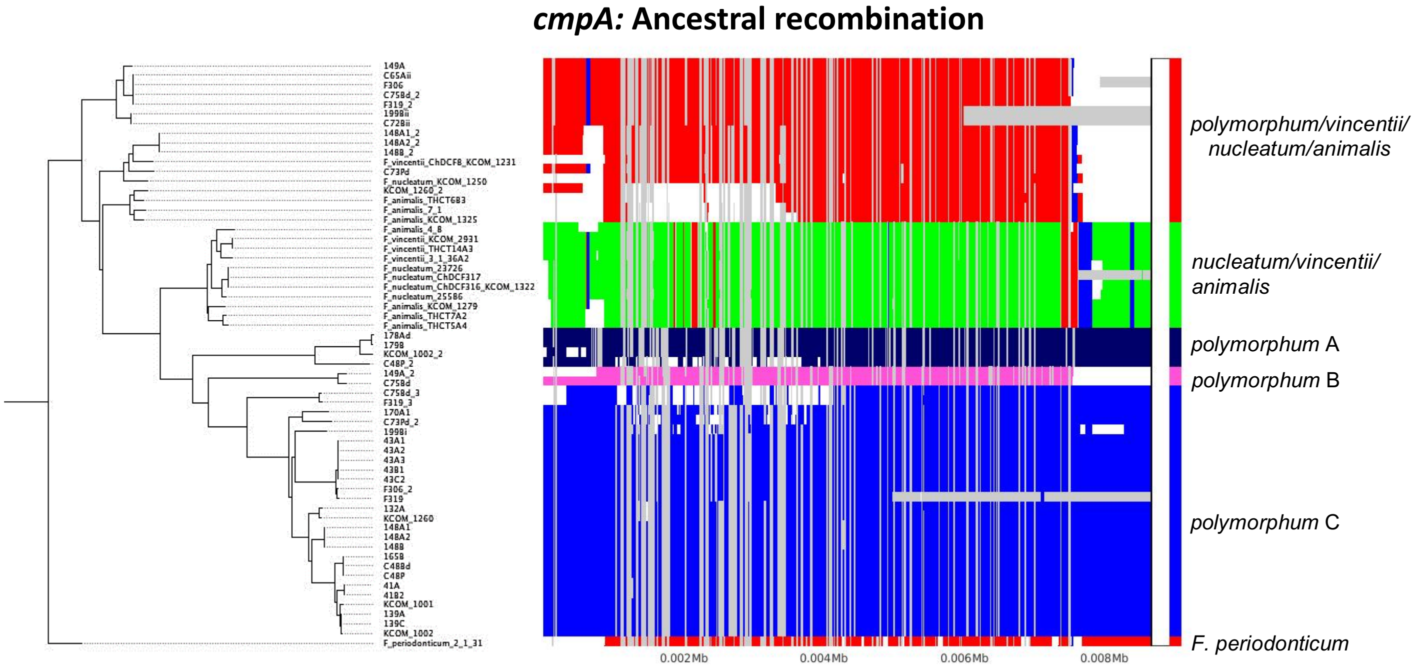

Figure S22. Analysis of recent recombination within the *cmpA* gene in *Fusobacterium* species using fastGEAR. Sequences of the *cmpA* gene from *F. periodonticum* and the indicated strains of *F. nucleatum* were aligned in MAFFT (v7.0) and subjected to analysis in fastGEAR to detect evidence of recombination. On the y-axis are the 61 sequences included in the sequence alignment ordered by position in a phylogenetic tree. The x-axis corresponds to the sequence positions of the respective genes. Regions in grey represent gaps in the alignment. The coloured legend on the right side of the panel shows division of the strains into lineages indicated by different colours.

Figure S23

cmpA: **Recent recombination**

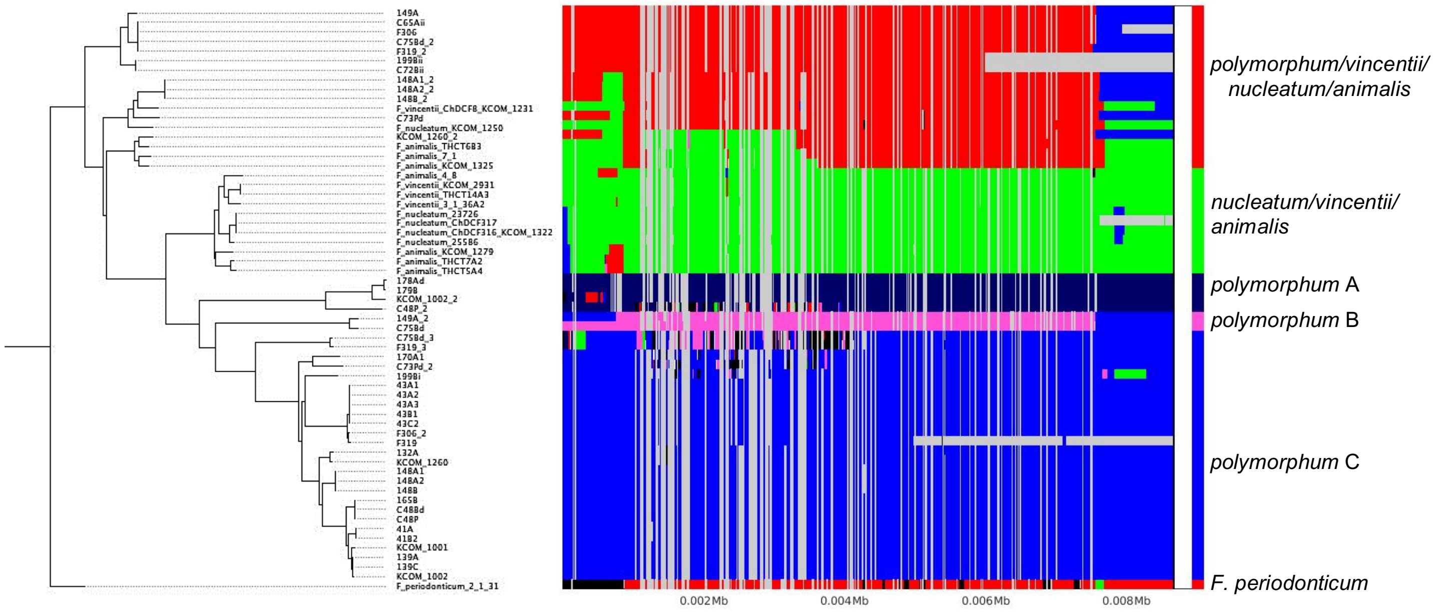

Figure S23. Analysis of ancestral recombination within the *cmpA* gene in *Fusobacterium* species using fastGEAR. Sequences of the *cmpA* gene from *F. periodonticum* and the indicated strains of *F. nucleatum* were aligned in MAFFT (v7.0) and subjected to analysis in fastGEAR to detect evidence of recombination. On the y-axis are the 61 sequences included in the sequence alignment ordered by position in a phylogenetic tree. The x-axis corresponds to the sequence positions of the respective genes. Regions in grey represent gaps in the alignment. The coloured legend on the right side of the panel shows division of the strains into lineages indicated by different colours. White sections represent recent recombination shown in Fig S23.

Figure S24

fusolisin: **Recent recombination**

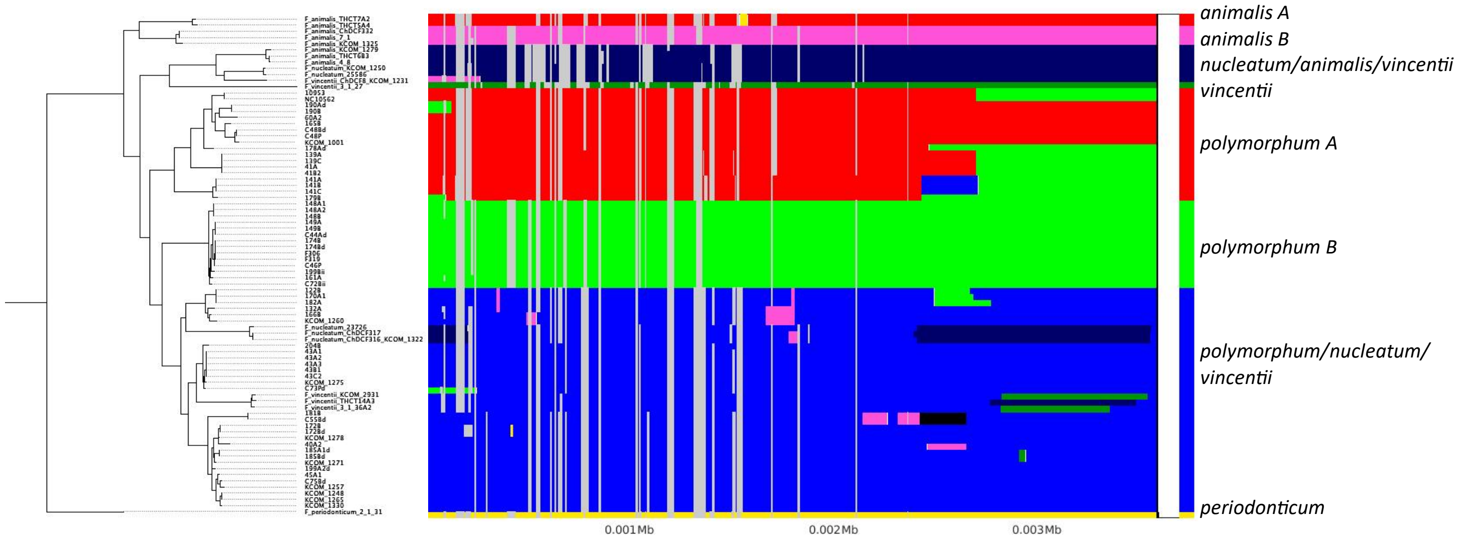

Figure S24. Analysis of recent recombination within the fusolisin gene in *Fusobacterium* species using fastGEAR. Sequences of the fusolisin gene from *F. periodonticum* and the indicated strains of *F. nucleatum* were aligned in MAFFT (v7.0) and subjected to analysis in fastGEAR to detect evidence of recombination. On the y-axis are the 81 sequences included in the sequence alignment ordered by position in a phylogenetic tree. The x-axis corresponds to the sequence positions of the respective genes. Regions in grey represent gaps in the alignment. The coloured legend on the right side of the panel shows division of the strains into lineages indicated by different colours.

Figure S25

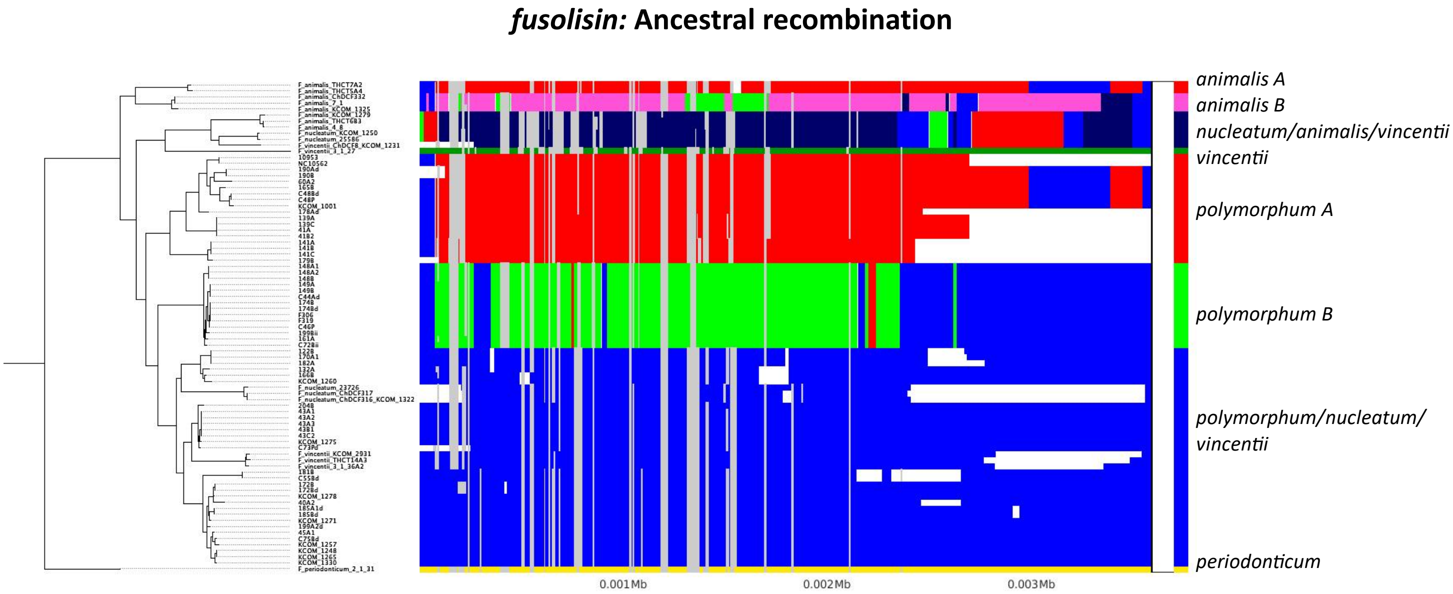

Figure S25. Analysis of ancestral recombination within the fusolisin gene in *Fusobacterium* species using fastGEAR. Sequences of the fusolisin gene from *F. periodonticum* and the indicated strains of *F. nucleatum* were aligned in MAFFT (v7.0) and subjected to analysis in fastGEAR to detect evidence of recombination. On the y-axis are the 81 sequences included in the sequence alignment ordered by position in a phylogenetic tree. The x-axis corresponds to the sequence positions of the respective genes. Regions in grey represent gaps in the alignment. The coloured legend on the right side of the panel shows division of the strains into lineages indicated by different colours. White sections represent recent recombination shown in Fig S25.

Figure S26

fvcE: **Recent recombination**

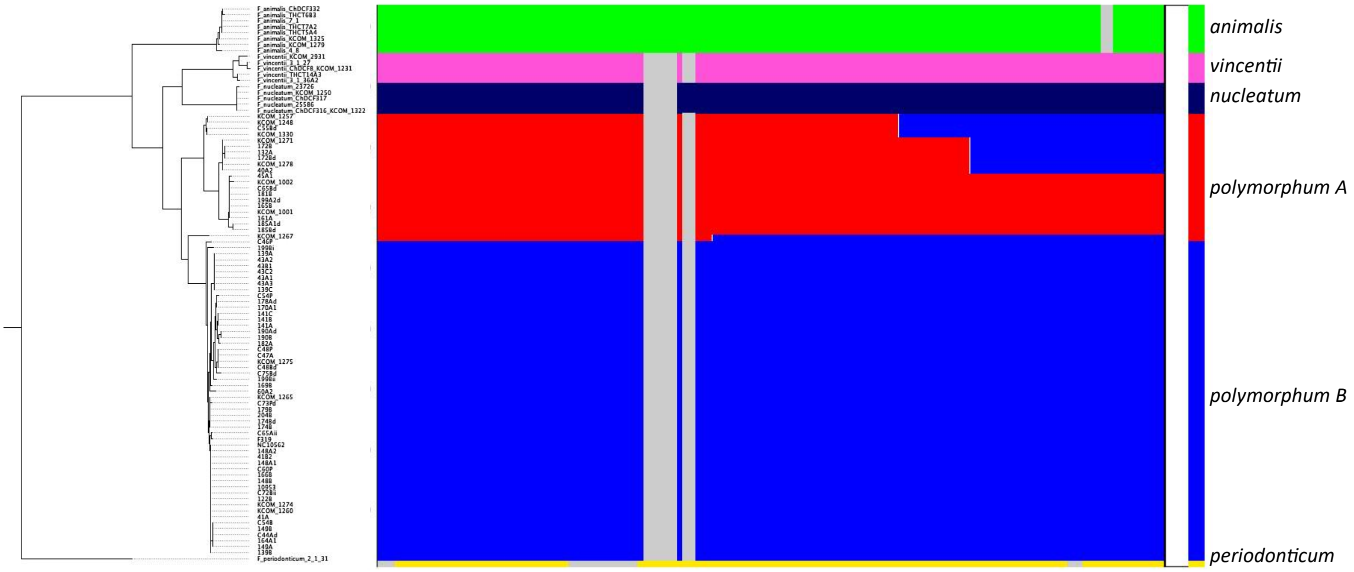

Figure S26. Analysis of recent recombination within the *tvcE* gene in *Fusobacterium* species using fastGEAR. Sequences of the *tvcE* gene from *F. periodonticum* and the indicated strains of *F. nucleatum* were aligned in MAFFT (v7.0) and subjected to analysis in fastGEAR to detect evidence of recombination. On the y-axis are the 93 sequences included in the sequence alignment ordered by position in a phylogenetic tree. The x-axis corresponds to the sequence positions of the respective genes. Regions in grey represent gaps in the alignment. The coloured legend on the right side of the panel shows division of the strains into lineages indicated by different colours.

Figure S27

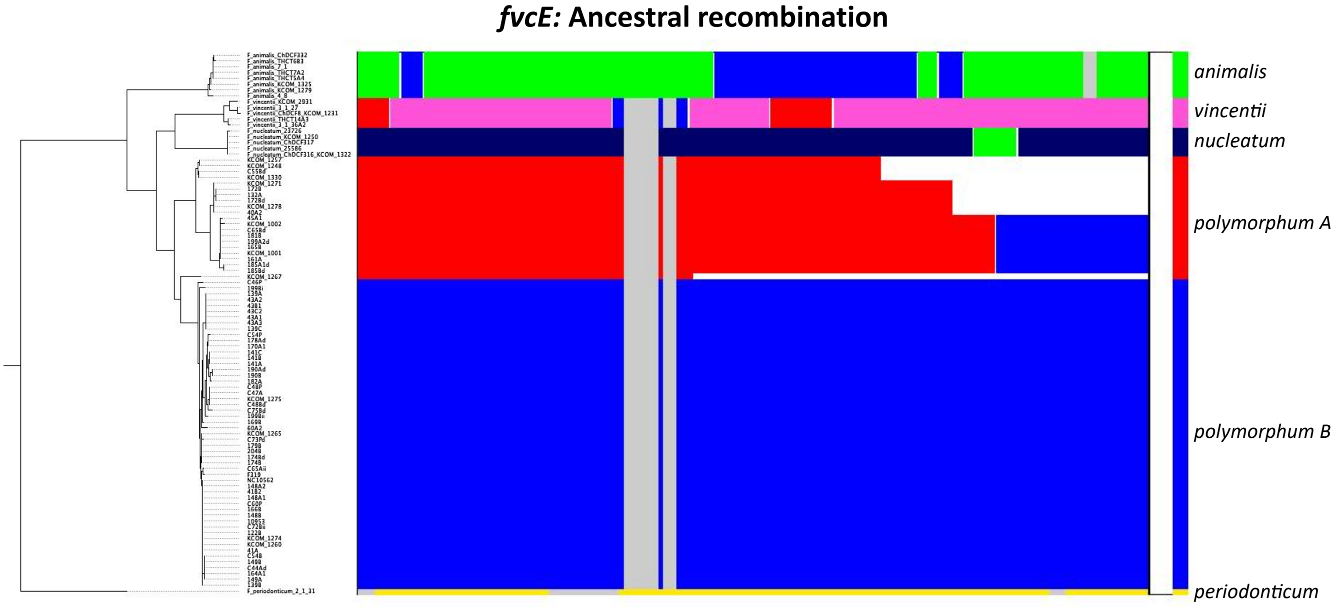

Figure S27. Analysis of ancestral recombination within the *tvcE* gene in *Fusobacterium* species using fastGEAR. Sequences of the *tvcE* gene from *F. periodonticum* and the indicated strains of *F. nucleatum* were aligned in MAFFT (v7.0) and subjected to analysis in fastGEAR to detect evidence of recombination. On the y-axis are the 93 sequences included in the sequence alignment ordered by position in a phylogenetic tree. The x-axis corresponds to the sequence positions of the respective genes. Regions in grey represent gaps in the alignment. The coloured legend on the right side of the panel shows division of the strains into lineages indicated by different colours. White sections represent recent recombination shown in Fig S27.

Figure S28

***fadA:* Recent recombination**

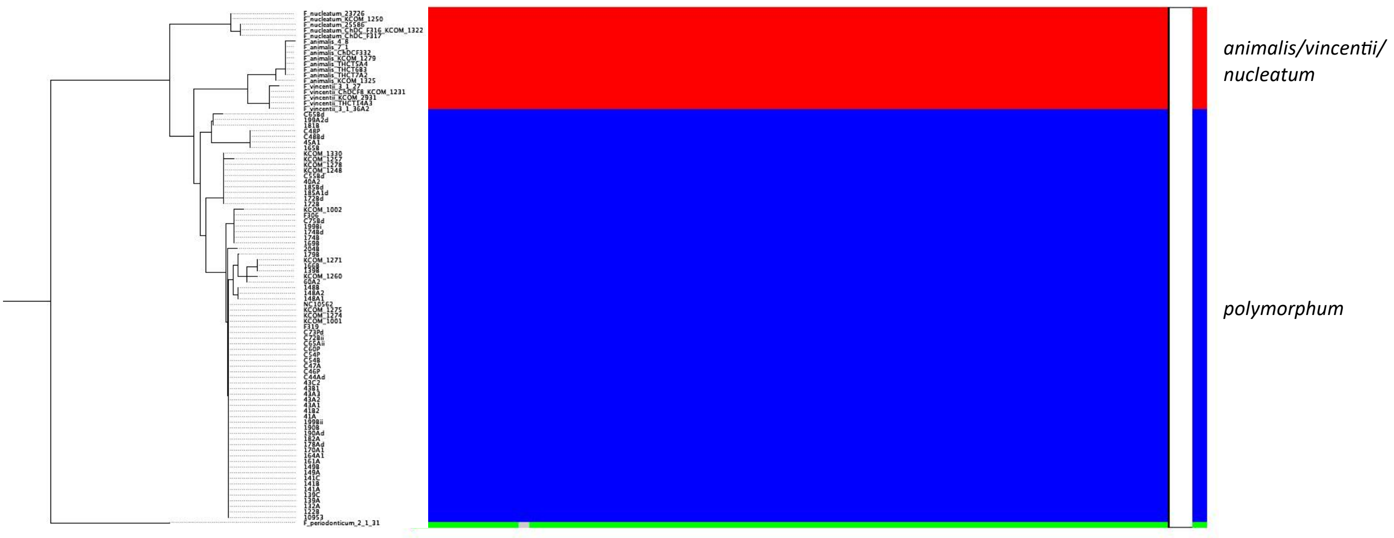

Figure S28. Analysis of recent recombination within the *fadA* gene in *Fusobacterium* species using fastGEAR. Sequences of the *fadA* gene from *F. periodonticum* and the indicated strains of *F. nucleatum* were aligned in MAFFT (v7.0) and subjected to analysis in fastGEAR to detect evidence of recombination. On the y-axis are the 92 sequences included in the sequence alignment ordered by position in a phylogenetic tree. The x-axis corresponds to the sequence positions of the respective genes. The coloured legend on the right side of the panel shows division of the strains into lineages indicated by different colours.

Figure S29

***fadA:* Ancestral recombination**

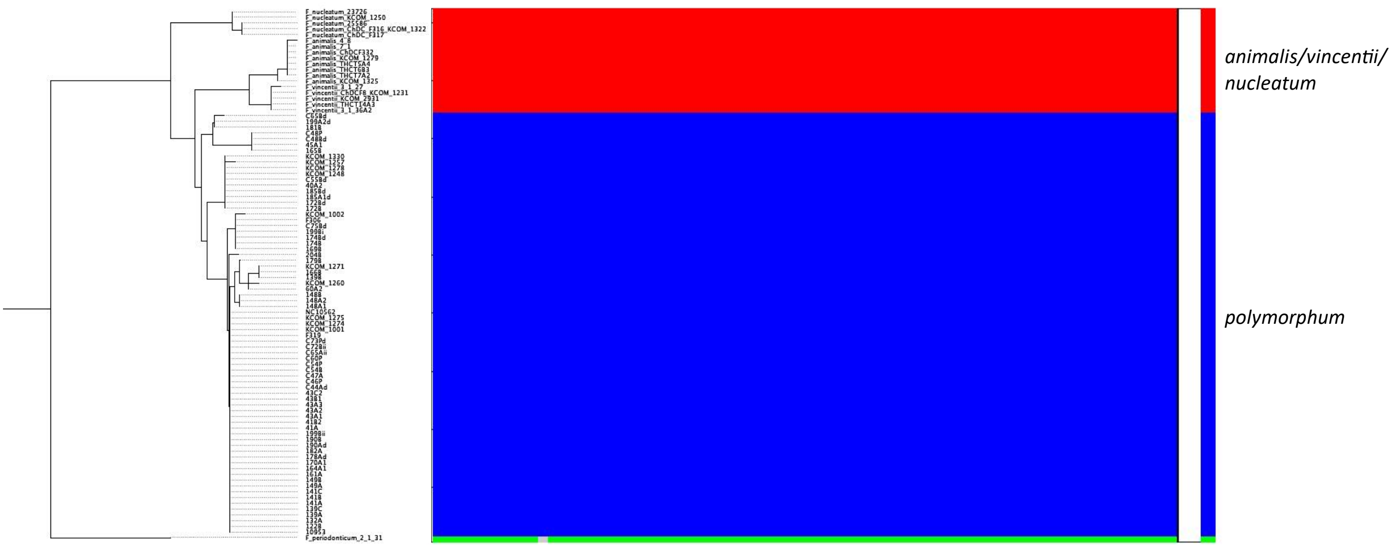

Figure S29. Analysis of ancestral recombination within the *fadA* gene in *Fusobacterium* species using fastGEAR. Sequences of the *fadA* gene from *F. periodonticum* and the indicated strains of *F. nucleatum* were aligned in MAFFT (v7.0) and subjected to analysis in fastGEAR to detect evidence of recombination. On the y-axis are the 92 sequences included in the sequence alignment ordered by position in a phylogenetic tree. The x-axis corresponds to the sequence positions of the respective genes. The coloured legend on the right side of the panel shows division of the strains into lineages indicated by different colours.
